# Comparative Study on the Crack Formation Tendency of the Laser Lap-Welded Steels with GA, GI and ZAM Coatings

**DOI:** 10.3390/ma19194082

**Published:** 2026-09-24

**Authors:** Chengjie Liu, Shiqin Su, Jiayi Zhang, Hua Pan, Yulai Gao

**Affiliations:** 1 State Key Laboratory of Materials for Advanced Nuclear Energy, Shanghai University, Shanghai 200444, China; liuchengjie@baosteel.com (C.L.); 1473969742@shu.edu.cn (S.S.); zjy17367553208@shu.edu.cn (J.Z.); 2 School of Materials Science and Engineering, Shanghai University, Shanghai 200444, China; 3 Automobile Steel Research Institute, R&D Center, Baoshan Iron & Steel Co., Ltd., Shanghai 201900, China

**Keywords:** laser lap welding, galvanizing coating, galvannealing coating, zinc-aluminum-magnesium coating, lateral crack, central crack

## Abstract

Steels coated with Zn-based coatings have been extensively employed in the manufacturing industry due to their excellent corrosion resistance. Unfortunately, crack defects can occur during the laser welding process. Three kinds of coatings were involved in the present study, namely galvannealing (GA), galvanizing (GI) and zinc–aluminum–magnesium (ZAM) coatings. To investigate the influence of different coatings (namely GA, GI and ZAM) on crack formation tendency, we carried out a comparative study on laser lap-welded joints with similar coating combinations (including Steel A (GA)/Steel A (GA), Steel B (GI)/Steel B (GI) and Steel C (ZAM)/Steel C (ZAM)) and dissimilar coating combinations (including Steel A (GA)/Steel C (ZAM) and Steel B (GI)/Steel C (ZAM)). The results showed that the joints with different zinc-based coatings exhibited distinct crack behaviors. Only the similar ZAM/ZAM joints revealed both lateral and central cracks, while the dissimilar GA/ZAM and GI/ZAM joints produced lateral cracks in the zones adjacent to the heat affected zone (HAZ). In particular, all the lateral cracks existed in the dissimilar joints, initiated and distributed on the ZAM-coated side. The microstructure and coating melting characteristics were analyzed in the process of crack initiation and propagation. It was considered that the lower melting temperature of ZAM (about 349 °C) compared with GA (about 423 °C) and GI (about 425 °C) could prolong the time to hold the melt status of the coating, accelerating the Fe dissolution, Al-Zn eutectic reaction (inducing lateral cracks) and liquid Zn penetration along the grain boundaries (causing central cracks), thereby increasing the crack susceptibility of the laser lap welding ZAM-coated steel plates.

## 1. Introduction

As the demands for corrosion resistance in metallic materials keep escalating in downstream application sectors including household appliances, photovoltaics, construction and automobiles, conventional bare steel sheets have failed to meet the crucial requirements for long-term stable and reliable service in complex and harsh working environments [1,2]. To face this challenge, a variety of metallic protective coatings have been continuously investigated, developed and iteratively optimized [3]. In the field of corrosion protection for steels, the mainstream coatings widely adopted in current engineering applications mainly consist of three typical categories: hot-dip galvannealing (GA), galvanizing (GI), and zinc–aluminum–magnesium (ZAM) coatings [4,5]. Relying on the refined compositional design and precise regulation of microstructures, the above-mentioned coatings can form compact and continuous protective layers on the surface of steel substrates [6]. These layers can not only exert exceptional physical barrier and shielding effects, but also achieve efficient electrochemical protection for the steel substrates via the sacrificial anode protection mechanism, thereby substantially prolonging the corrosion-resistant service life of metallic materials in various environments [7].

Serving as the most predominant corrosion protection strategies in the field of steel, GI and GA coatings were applied on a large scale in industrial sectors for decades attributing to their mature fabrication processes and stable protective performance [8]. In contrast, ZAM-coated steel has become a preferred alternative to those traditional GI/GA steels. The preference is rooted in ZAM-coated steel’s markedly enhanced corrosion resistance, edge self-healing performance and lubricating properties [9,10]. ZAM coatings have evolved into multiple mature systems with low-aluminum, medium-aluminum and high-aluminum contents. As a result, relevant researches in this field gradually extended from fundamental compositional design and microstructure manipulation to the core research fields covering coating service performance and processing formability adaptability [11,12,13,14].

The microstructural discrepancies among different coating systems directly determined the distinct divergence of their core corrosion protection mechanisms and corrosion resistance. GI coating featured a single η-Zn phase as the dominant microstructure, and mainly achieved long-term protection for steel substrates via the sacrificial anode effect of zinc [15]. Nevertheless, such coatings exhibited apparent application drawbacks: this kind of coating could be corroded at a relatively rapid rate in harsh corrosive environments with high humidity and high chloride content. Moreover, GI coating exhibited extremely limited targeted protective capacity for exposed areas of steel substrates such as cuts and scratches [16]. GA coatings were subjected to alloying annealing treatment, forming Fe-Zn intermetallic compound layers dominated by Γ (Fe_3_Zn_10_) and δ (FeZn_7_–_10_) phases [17,18]. Although this process effectively improved the high-temperature service stability of the coatings, it simultaneously reduced the sacrificial anode electrochemical activity of the coatings, making them prone to local pitting corrosion and posing significant localized corrosion risks during long-term service [19]. In contrast, ZAM coatings achieved a magnitude-scale enhancement in corrosion resistance via the synergistic effect of the Al and Mg elements, and their microstructure consisted mainly of Zn-rich primary phases, Zn-Al binary eutectics and Zn-Al-Mg_2_Zn_11_ ternary eutectics [20,21,22]. The synergistic incorporation of Mg and Al into ZAM coatings facilitated the formation of dense, stable corrosion products that effectively impeded the penetration of corrosive media, thereby imparting outstanding self-healing properties to the coatings at steel substrate cut edges [23,24,25,26].

However, steels with GA, GI and ZAM coatings were highly susceptible to defects including porosity, spatter and cracks during the welding process [27,28,29]. Among all observed weld defects, liquid metal embrittlement (LME) cracking represents one of the typical cracking phenomena in steels coated with GA, GI and ZAM coatings [30]. The LME phenomenon was widely presented in mainstream welding processes such as resistance spot welding (RSW) and laser welding (LW) of coated advanced high-strength steels [31,32,33]. It has become one of the core technical bottlenecks to restrict the achievement of efficient and reliable connections in those coated steels [34,35]. Razmpoosh et al. [36] explored the LME susceptibility associated with laser welding of galvanized 22MnB5 boron steel and demonstrated that increased heat input prolonged the high-temperature residence time, which further accelerated the Zn diffusion and propagation of LME cracks. In our previous study, the LME susceptibility of GI and EG coated DP1180 steels during RSW was investigated, revealing the critical role of the interfacial oxide layer in mitigating LME [37]. Yu et al. [30] compared the LME susceptibility of ZAM and GI coatings on the dual phase steel with 1000 MPa grade via hot tensile and RSW tests, and found that elevated Al content in the ZAM coating exerted an adverse effect on LME susceptibility.

This study emphasized the formation behavior of welding cracks in different zinc-based coated steels. Three typical coated steel sheets (Steel A (QP1180+GA), Steel B (QP980+GI) and Steel C (DC53D+ZAM)) were employed as the raw experimental materials for comparative laser lap welding tests. The macromorphological characteristics of cracks and interfacial element distribution in the welded joints were systematically analyzed by optical microscopy (OM) and scanning electron microscopy (SEM). By analyzing and comparing the crack length and distribution, the trends in cracking behavior and the associated formation mechanisms for GA, GI and ZAM coatings during laser lap welding were elucidated.

## 2. Materials and Methods

Three typical steels coated with Zn-based coatings were employed as experimental materials, namely ultra-high-strength galvanized steel (Steel A (GA)), ultra-high-strength galvanized steel (Steel B (GI)), and low-carbon steel with zinc–aluminum–magnesium coating (Steel C (ZAM)). The chemical composition of these base metals is listed in Table 1. The GI coating employed in the experiments consisted predominantly of pure zinc as its matrix phase, with a zinc content exceeding 99.8 wt.% and trace aluminum (0.18–0.20 wt.%) incorporated into the coating. The Fe content in the GA coating ranged from 9 wt.% to 13 wt.%, with trace aluminum (0.125–0.135 wt.%) introduced into the zinc bath to inhibit the abnormal growth of brittle Fe-Zn intermetallic compound layers. The ZAM coating was Zn-6Al-3Mg, with zinc as the matrix and alloying modification achieved via the combined addition of 6 wt.% Al and 3 wt.% Mg.

Three typical Zn-based coated steels were chosen as the experimental materials, namely ultra-high-strength galvannealed steel (Steel A, QP1180+GA), ultra-high-strength galvanized steel (Steel B, QP980+GI), and low-carbon Zn-Al-Mg coated steel (Steel C, DC53D+ZAM). The measured yield strength, tensile strength and fracture elongation of these three material systems are listed in Table 2. The steel substrates were coated on both sides with coating masses of 45 g/m^2^ (GA), 60 g/m^2^ (GI), and 90 g/m^2^ (ZAM) per side. Their average single-side coating thicknesses were approximate 6.2 μm, 8.4 μm and 13.2 μm, respectively.

The laser welding experiments were performed using a TruDisk-5001 solid-state laser (Trumpf GmbH, Ditzingen, Germany) with a continuous mode. The dimension of each test specimen was 45 mm in width and 120 mm in length. A 25 mm-length area was welded at the center of each specimen. Prior to welding, the sheet surfaces were cleaned with alcohol to remove surface oil contamination. Three parallel specimens were welded for each group under identical processing parameters. After welding, one cross-sectional specimen was prepared at the center of each welded joint for microscopic observation.

All welded specimens employed in this study were fabricated in a lap joint configuration, covering both similar and dissimilar coating combinations for the laser welding investigation. The lower sheet was placed on the welding platform and four supports with the thickness of 0.1 mm were inserted on the surface of the lower sheet to control the gap of 0.1 mm. This gap was designed to facilitate the escape of zinc vapor during the welding process. The upper sheet was lapped on the lower sheet and clamped by fixtures to prevent displacement during welding. A schematic of the welding setup is shown in Figure 1. During the welding, the laser beam was vertically irradiated from the upper sheet side and both sheets were melted and jointed to be a whole joint.

Three similar coating combinations, namely Steel A (GA)/Steel A (GA), Steel B (GI)/Steel B (GI), and Steel C (ZAM)/Steel C (ZAM), were designated as specimens S1 to S3, respectively. Two dissimilar coating combinations, consisting of Steel A (GA)/Steel C (ZAM) and Steel B (GI)/Steel C (ZAM), were labeled as S4 and S5 in sequence. Detailed sheet thicknesses and lap sequences corresponding to each combination are presented in Table 3. Subsequently, welding parameters were determined based on the total sheet thickness of each group, with full penetration of the lower sheet set as the welding control criterion. Such penetration control was achieved solely by tuning the laser power, without any other welding parameter adjustment. The laser powers allocated for joint combinations S1 to S5 were 4.0 kW, 4.5 kW, 4.75 kW, 4.5 kW and 4.75 kW, respectively. Differentiated laser power settings were adopted for different welding combinations, which was formulated as a process matching design based on the total thickness of the base sheets. This design principle was intended to ensure complete joint penetration while simultaneously avoiding possible welding defects, e.g., burn-through induced by excessive heat input. The other welding parameters were kept unchanged during the experiment. The welding speed for all welded combinations was set at 3 m/min and the defocusing distance was uniformly 25 mm. All laser welding tests were conducted in an atmospheric environment; no shielding gas was applied during the welding process.

After welding, a cross-section was cut perpendicular to the welding direction of the joint and mounted to prepare a standard metallographic specimen. Following mechanical polishing, the unetched surface morphology was first observed and recorded using optical microscopy (OM, VHX-S770, Keyence Corporation, Osaka, Japan). The polished specimen was then etched with a 4 vol.% nitric solution and the metallographic image of the welded joint was subsequently examined via OM. Upon completion of metallographic characterization, scanning electron microscopy (SEM, ZEISS EVO MA25, Carl Zeiss AG, Oberkochen, Germany) coupled with energy dispersive spectroscopy (EDS, X-MaxN 20, Oxford Instruments, Abingdon, UK) was utilized to systematically investigate the micromorphology and elemental distribution within the defective regions of the welded joint. The scanning electron microscope (SEM) was operated at an accelerating voltage of 20 kV with a working distance of approximately 5 mm. For EDS data acquisition, the acquisition time was set to 20 s.

The finite element model was established in MSC.Marc 2023.3 software to simulate the laser lap welding process. A conical heat source was adopted in this transient thermal simulation to replicate the real geometry of the fusion zone. Temperature-variant thermal properties and boundary conditions covering heat conduction, convection and thermal radiation, were incorporated into the numerical model. The thermal conductivity, specific heat capacity and density of the materials were calculated using JMatPro Version 7.0 software and the corresponding data were plotted in Figure 2.

The heat conduction process within the welded specimen during welding was governed by the nonlinear heat conduction equation. Heat exchange between the specimen and the surrounding environment was taken into account, and the heat dissipation process was described by a combination of convective heat transfer and radiative heat transfer. Convective heat exchange between the specimen and the ambient environment was modeled by Newton’s law of cooling, with the convective heat transfer coefficient set to 2.0 × 10^−5^ W·mm^−2^·°C^−1^. Radiative heat loss from the specimen surface was calculated according to the Stefan–Boltzmann law, with the Stefan–Boltzmann constant adopted as 5.67 × 10^−12^ W/(mm^2^·K^4^). For the temperature field simulation, both the initial temperature of the specimen and the ambient temperature were set to 20 °C. The welding parameters used in the simulation were fully consistent with those employed in the joints in the experiments.

## 3. Results and Discussion

### 3.1. Crack Observation of Laser Lap-Welded Joints with Similar Coating Combinations

To investigate the presence of crack defects in laser lap-welded joints of GA coated steel, observations and analysis were performed on the Steel A (GA)/Steel A (GA) laser lap-welded joint (Specimen No. S1). Figure 3 shows the cross-sectional metallographic micrograph of specimen S1 in unetched condition. Figure 3a presented the macroscopic cross-sectional morphology of the joint, and no visible welding defects were found by macroscopic observation. In our previous study [38], we found that crack defects tended to form at the interface of lap joints. Therefore, further high-magnification microstructural characterization was performed on Zones A and B, which are marked in Figure 3a. Detailed observation revealed that no welding cracks were detected in the inspected sections of either Zone A or Zone B (see Figure 3b,c).

Figure 4 displayed the cross-sectional metallographic images of the Steel B (GI)/Steel B (GI) laser lap-welded joint corresponding to specimen combination S2 in unetched condition. Figure 4a shows the macroscopic cross-sectional morphology of the joint. No obvious welding defects were detected in the inspected section. Similarly, high-magnification microscopic observation was carried out on the two critical regions (Figure 4b,c) and the results demonstrated that no welding cracks were observed in the inspected sections of either of the regions.

This work also aims to clarify the characteristics of welding cracks in laser lap-welded joints of ZAM-coated steel. To realize this objective, the Steel C (ZAM)/Steel C (ZAM) joint designated as Specimen No. S3 was adopted. Figure 5 presents the cross-sectional metallographic micrograph of the S3 joint under the unetched condition. Figure 5a shows the macroscopic cross-sectional morphology of the joint. The corresponding high-magnification microstructures are displayed in Figure 5b,c. Further observations of the morphology and distribution of cracks demonstrated the presence of two distinct crack types within the joint. The first type, with a maximum length (*l*_2_) of 35 μm, propagated along the sheet thickness direction (see Figure 5b,c). The second type attained a maximum length (*l*_1_) of 315 μm and extended horizontally within the weld seam (see Figure 5b).

To clearly reveal the microstructural characteristics of each region of the joint and further verify the presence of fine crack defects, specimen S1 was subjected to an etching treatment followed by subsequent metallographic observations and analysis. The cross-sectional metallographic images after being etched could clearly distinguish the microstructural differences among the weld zone (WZ), heat affected zone (HAZ), and base metal zone (BM). Specifically, high-magnification observations on these two typical defect-prone regions (Zone A and Zone B, marked earlier) indicated that no welding cracks appeared (see Figure 6).

To further clarify the microstructural characteristics of the GI coated laser lap-welded joint, specimen S2 was subjected to an etching treatment. Consistent with the aforementioned research strategy, high-magnification microstructural characterization was further performed on the two critical regions examined under the unetched condition. The results indicated that no welding cracks could be detected in the above regions, which was in good agreement with the observations obtained from the GA coated joint (see Figure 7).

Figure 8 presents a metallographic image of the chemically etched laser lap-welded joint fabricated with the ZAM-coated similar sheet combination (Specimen S3). The key observation regions were consistent with those in Figure 3, facilitating the comparative analysis of microstructural characteristics and defect distribution in the etched joint. Further observation of the crack morphology and distribution location revealed that the cracks in the joint could be classified into two categories. One type of crack was distributed adjacent to the heat affected zone (HAZ), and this crack propagated into the base metal matrix (see Figure 8b,c). According to its distribution location and propagation morphology, it was defined as a lateral crack. The other type of crack was mainly located at the center of the weld fusion zone and propagated approximately horizontally (see Figure 8b). According to its morphological features and distribution location, this crack was identified as a central crack.

Based on the above welding analysis results, significant differences were observed in the crack formation tendencies among the three types of laser lap-welded joints with similar coatings, namely Steel A (GA)/Steel A (GA), Steel B (GI)/Steel B (GI), and Steel C (ZAM)/Steel C (ZAM). Among these joints, crack defects were generated only in the coated Steel C (ZAM) joint during welding. The cracks formed in this type of joint could be classified into two typical morphologies: lateral cracks and central cracks.

### 3.2. Crack Observation of Laser Lap-Welded Joints with Dissimilar Coating Combinations

To systematically investigate the crack initiation characteristics of laser lap-welded joints with dissimilar coating combinations, the Steel A (GA)/Steel C (ZAM) laser lap-welded joint (Specimen No. S4) was selected as the research object for microstructural observation and defect characterization. Figure 9 shows the cross-sectional metallographic micrograph of specimen S4 in the unetched condition. As presented in Figure 9a, the macroscopic cross-sectional morphology of the joint exhibited obvious signs of welding defects, which was in sharp contrast to the defect-free observation result of the similar GA coated joint discussed above. Consistent with the aforementioned research scheme, high-magnification microstructural observation and analysis were further performed on Zones A and B, as marked in Figure 9a. The high-magnification observations indicated that no crack defects were detected in the inspected sections of the GA coated side, whereas obvious cracks were observed on the ZAM-coated side. According to its distribution location and propagation morphology, this crack was a lateral crack.

To characterize the welding crack susceptibility of GI/ZAM dissimilar coating combination, this study selected the Steel B (GI)/Steel C (ZAM) laser lap-welded joint (Specimen No. S5) for experimental observation and analysis. Figure 10 displays the cross-sectional metallographic images of specimen S5 in the unetched condition. Figure 10a shows the macroscopic cross-sectional morphology of the joint, where obvious welding defects were observed inside the joint, which was in sharp contrast to the observation result of no obvious defects in the GI similar coated joint discussed above. Similarly, high-magnification microscopic observation was carried out on the two critical regions (Figure 10b,c). The results demonstrated that no crack defects were detected in the inspected sections of the GI coated side. However, a crack could be clearly observed on the ZAM-coated side. According to the crack type definition established earlier, this crack was identified as a lateral crack.

To further clarify the microstructural characteristics of the laser lap-welded joint with GA/ZAM dissimilar coatings (Specimen No. S4), the specimen was subjected to chemical etching treatment. The etching treatment clearly highlighted the microstructural boundaries among the weld zone, heat affected zone and base metal. Similarly, high-magnification microscopic observations were performed on the two critical regions (Zone A and Zone B) marked in Figure 11a (see Figure 11b,c). Lateral crack defects were detected in both critical regions, and the cracks exhibited a distinct regional distribution. All lateral cracks were only present near the HAZ on the ZAM-coated side and exhibited a characteristic of propagating along the weld fusion line in the sheet thickness direction.

Figure 12 showed the microstructural characteristics of the chemically etched laser lap-welded joint with GI/ZAM dissimilar coatings (Specimen No. S5). Consistent with the mentioned method, high-magnification microscopic observation was carried out on the two critical regions (Zone A and Zone B) marked in Figure 12a (see Figure 12b,c). The distribution location and propagation morphology of these cracks were similar to those observed in the above-mentioned GA+ZAM dissimilar coating joints.

Based on the analysis of the comprehensive welding joints, the two types of laser lap-welded joints with dissimilar coatings (Steel A (GA)/Steel C (ZAM) and Steel B (GI)/Steel C (ZAM)) exhibited essentially consistent crack formation characteristics. Only lateral cracks were observed in both joints, and all such cracks initiated and distributed on the side of the Steel C (ZAM) coated steel.

The original ZAM coating on the DC53D+ZAM steel surface was mechanically removed. Laser welding experiments were subsequently performed under the same welding conditions. Figure 13 shows the metallographic images of DC53D+ZAM (ZAM coating removed)/DC53D+ZAM (ZAM coating removed) laser lap-welded joints. It was found that no cracks could be observed in the inspected sections of the welded joint under the identical welding conditions after coating removal. Accordingly, it was inferred that the cracks detected in the DC53D+ZAM laser lap-welded joint were primarily induced by the ZAM coating.

### 3.3. Effect of Coating Types on Welding Crack

Figure 14 presents the measured average crack lengths of laser lap-welded joints S1–S5. Statistical analysis of the two similar coating joints (S1–S2) showed that no obvious welding crack defects were observed in the inspected sections of either the QP1180+GA or the QP980+GI similar coating joint. However, two typical crack defects were detected in the DC53D+ZAM similar coating joint (S4). The average length of the lateral cracks was about 22 μm (7 cracks observed), while that of central cracks reached approximate 255 μm (5 cracks observed).

For those two dissimilar joint combinations, namely S4 (QP1180+GA/DC53D+ZAM) and S5 (QP980+GI/DC53D+ZAM), statistical analysis revealed that no central crack defects were detected in the inspected sections of either welded joint. Only lateral cracks were observed in the observation regions. Furthermore, the measured lengths of lateral cracks differed between these two joints. The average crack length of S4 was about 50 μm (6 cracks observed), and the average crack length of S5 was around 27 μm (5 cracks observed). The difference in lateral crack lengths between these two joints was possibly related to the slightly lower cooling rate of the S4 combination relative to the S5 combination.

The initiation mechanism of central cracks in the S3 joint combination was comparatively investigated, and the elemental distribution characteristics in the crack region as well as their corresponding effects on the crack formation were elucidated. Area mapping characterization was conducted on the central crack region of the joint using EDS to accurately obtain the elemental distribution information (Figure 15). The analytical results revealed that the observed region exhibited a significant enrichment of Zn, which was mainly distributed along the propagation path of the central crack (Figure 15b). However, no localized enrichment or segregation of elements such as Mg and Al was found along the entire crack propagation path (Figure 15c,d). These results further verify that the enrichment of Zn was the key factor responsible for the initiation of central crack in the weld zone. When combined with the element distribution results in Figure 15b,c, the central cracks were determined to be the results of Zn penetration along the boundaries of the welding zone.

Figure 16 presents the EDS elemental mapping analysis results of the lateral crack in the S5 joint. The analysis revealed that pronounced Zn enrichment existed within the lateral crack and Zn was distributed continuously along the crack region (Figure 16b). Meanwhile, distinct Al segregation was observed at the contact interface between the lateral crack and the base metal, leading to the formation of a continuous Al-enriched layer (Figure 16d). Furthermore, as shown in Figure 16c, no Mg enrichment was detected in the lateral crack region.

Although ternary phases containing Mg could possibly be formed within the Zn–6Al–3Mg coating, only Zn and Al were traced as the dominant elements associated with crack propagation, as revealed by the EDS mapping of the lateral cracks (Figure 16). The effect of elemental Mg and the formation of ternary phases were not demonstrated by the present results. Nevertheless, the potential contribution of Mg or ternary eutectic phases could not be fully excluded, and this aspect will be addressed in subsequent investigations.

To further probe the phase constitution within crack regions, micro-area XRD (Bruker D8 Discover) with a 20 μm spot diameter was additionally conducted. The corresponding XRD patterns are presented in Figure 17. Unfortunately, no useful phase information was obtained from the crack zones. This observation is the result of inadequate signal collection for the potentially existing phases, where target signals were masked by noise and a poor signal-to-noise ratio.

Based on the experimental results of joints with similar and dissimilar coatings (S1–S5), the ZAM coating exhibited a significant effect on crack formation in laser lap-welded joints. EDS elemental mapping analysis further confirmed that distinct enrichment of coating elements occurred inside the cracks, which directly indicated that the liquid metal formed by coating melting was the crucial factor to induce the initiation of central cracks and lateral cracks. Previous studies reported that the melting temperatures of the GA, GI and ZAM coatings were approximately 665 °C [39], 420 °C [40] and 380 °C [41], respectively. It was evident that the addition of Mg could reduce the melting temperature of the ZAM coating relative to the GA and GI coatings [42], and therefore extended the duration of the liquid state of the ZAM coating during the laser lap welding process. Meanwhile, the ZAM coating readily formed a lower-melting-point liquid phase.

On the other hand, the elements Al and Zn could produce a eutectic reaction, as shown in Equation (1) [43] and the Al-Zn binary phase diagram (Figure 18) [43].

(1) $$L\overset{382\;{}^{\circ}C}{⇀}\alpha \left(\mathrm{Al}\right)+\eta (\mathrm{Zn})$$

Based on the above analysis, the ZAM coating could be melted because of the heat input of the laser, and it remained molten for a relatively long time. Resultantly, the melted coating could penetrate and diffuse along the grain boundaries in the fusion zone of the joint to form the central cracks. On the other hand, the melted ZAM coating could maintain prolonged contact with the steel substrate, accelerating the reaction between the coating elements Al and Zn to induce the lateral cracks. Based on the above illustrated mechanism for the formation of central and lateral cracks, the rapid cooling of the welded joint by optimizing the welding process was expected to reduce or even prevent the production of these two kinds of defects.

To further illustrate the effects of different laser welding powers on various joint combinations, we present numerical simulation results of the welding temperature field (see Figure 19). The thermal cycle curves at the critical position (P1 in Figure 19a) prone to crack initiation for different joint configurations were comparatively analyzed. The cross-sectional temperature field contours during welding for different joint combinations were shown in Figure 19b. Overall, the regions with peak temperatures above 1500 °C for the S1–S5 joints were closely matched with the fusion zone of the corresponding as-welded joints, as predicted by the numerical simulation results in Figure 6a, Figure 7a, Figure 8a, Figure 11a and Figure 12a. Typical comparisons between experimental and numerical simulations for laser lap-welded joint S3 are presented in Figure 20, showing a reasonable similarity in fusion zone dimensions. Thus, the comparison results could demonstrate the reliability of the finite element model. It should be noticed that only minor differences existed in the thermal cycle processes adjacent to the critical region (Figure 19c). These results indicated that the slight differences in laser power parameters were not the dominant factor to account for the crack formation in the involved steel joints with different coatings.

**Figure 19:**
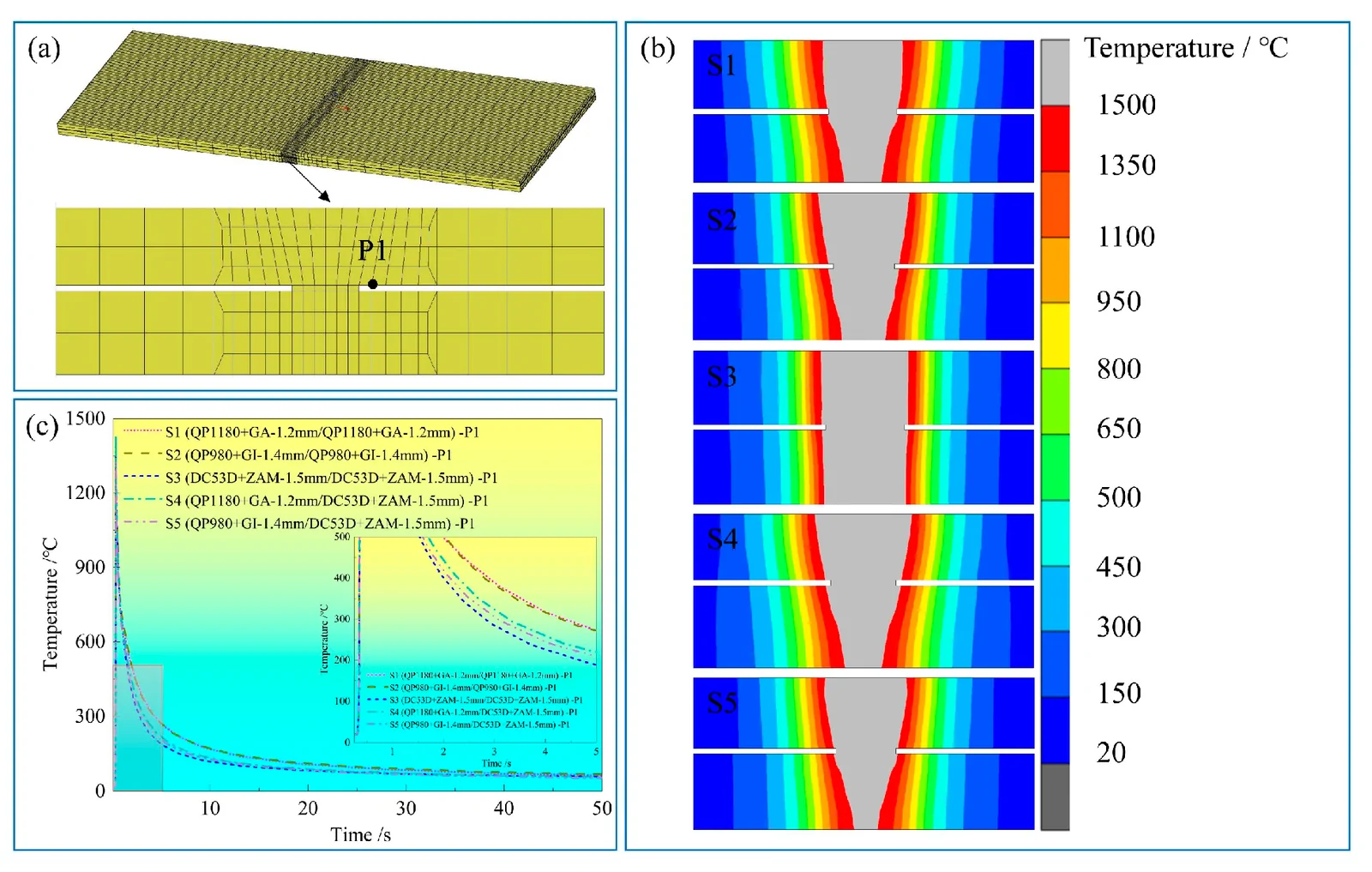
Finite element simulation analysis of the laser-welded lap joints: (**a**) meshed finite element model, (**b**) simulated temperature field distribution, (**c**) thermal cycle curves of the characteristic node P1.

Figure 21 showed the fast scanning calorimetry (FSC, Spark III, Functional Materials Rostock e.V., Rostock, Germany) curves of the GA, GI and ZAM coatings. The results revealed that the melting temperatures of the GA, GI and ZAM coatings were approximately 423 °C, 425 °C and 349 °C, respectively. The FSC thermal curve of the ZAM coating was shown in Figure 21c. A distinct endothermic peak was detected at 279 °C, which was induced by the inverse eutectoid solid-state transformation of the Zn-Al binary alloy system. This thermal behavior was in good agreement with the experimental results reported by Peter et al. [44]. Compared with those of GA and GI coatings, the melting temperature of the ZAM coating was effectively reduced to approximate 349 °C.

Among them, the ZAM coating exhibited an obvious lower melting temperature. Under the effect of welding thermal cycles, the low-melting ZAM coating remained in the liquid state for a longer duration, which was more likely to trigger interfacial interactions with the substrate and resultantly induce the crack defects. This evidence confirmed that the coating type itself could produce a crucial impact on the crack formation during laser lap welding.

In the S4 and S5 dissimilar laser lap-welded joints, the ZAM-coated sheet was positioned on the bottom, yet the GA and GI coated sheets were placed on the top. Accordingly, lateral cracks were observed to form exclusively on the ZAM side (the bottom). To elucidate the dependence of lateral crack formation on the type of coating rather than sheet stacking orientation, two additional reverse configurations of laser lap-welded joints were examined. These joints consisted of a ZAM coating on top/GA coating on bottom joint (the reverse of the S4 joint) and a ZAM coating on top/GI coating on bottom joint (the reverse of the S5 joint).

As for the reversed DC53D+ZAM/QP1180+GA joint, lateral cracks were detected on the DC53D+ZAM side (as shown in Figure 22), despite the DC53D+ZAM sheet being positioned as the top plate in this combination. An identical lateral cracking behavior was also identified in the reversed DC53D+ZAM/QP980+GI laser lap-welded joints (see Figure 23). These results demonstrated that the lateral cracking behavior of dissimilar laser lap-welded joints was governed by the coating type rather than the stacking sequence.

## 4. Conclusions

(1) Based on the welding results of laser lap-welded joints with similar coatings (Steel A (GA)/Steel A (GA), Steel B (GI)/Steel B (GI), and Steel C (ZAM)/Steel C (ZAM)), these three types of joints showed significant differences in crack formation characteristics. Lateral and central cracks were only detected in the ZAM/ZAM joints during the welding process.

(2) From the experimental results of laser lap joints with dissimilar coatings (Steel A (GA)/Steel C (ZAM) and Steel B (GI)/Steel C (ZAM)), lateral cracks were found in the HAZ of both joint combinations, and all such defects were exclusively initiated and distributed on the ZAM-coated steel.

(3) The melting temperatures of GA, GI and ZAM coatings were compared, and the results revealed that the ZAM coating possessed a lower melting temperature. The liquid metal formed by the melting of the ZAM coating exhibited a longer residence time under the laser welding process, which increased the tendency to trigger the two kinds of cracks. Based on the formation mechanism of these cracks, the rapid cooling of the welded joint was anticipated to reduce or even prevent crack formation by optimizing the welding process.

## Figures and Tables

**Figure 1:**
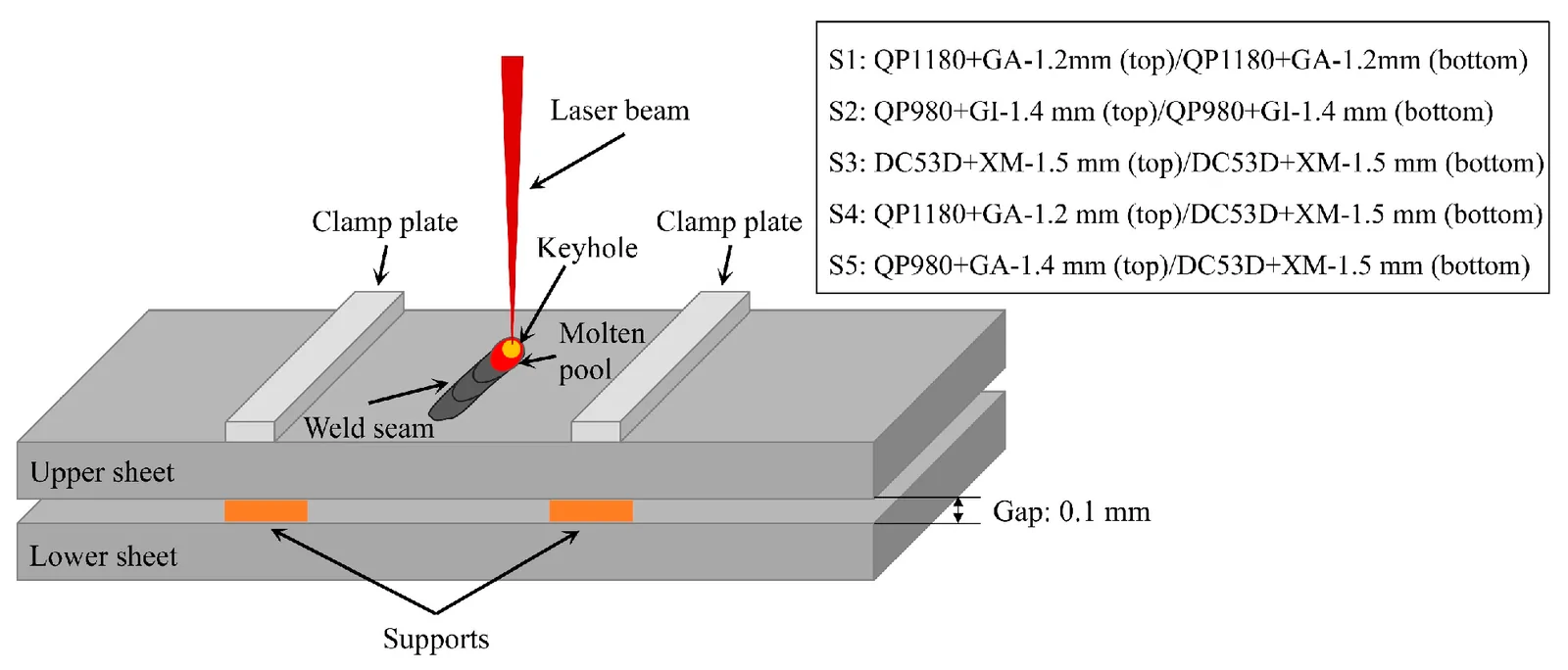
Schematic of the laser lap welding of the various joint combinations.

**Figure 2:**
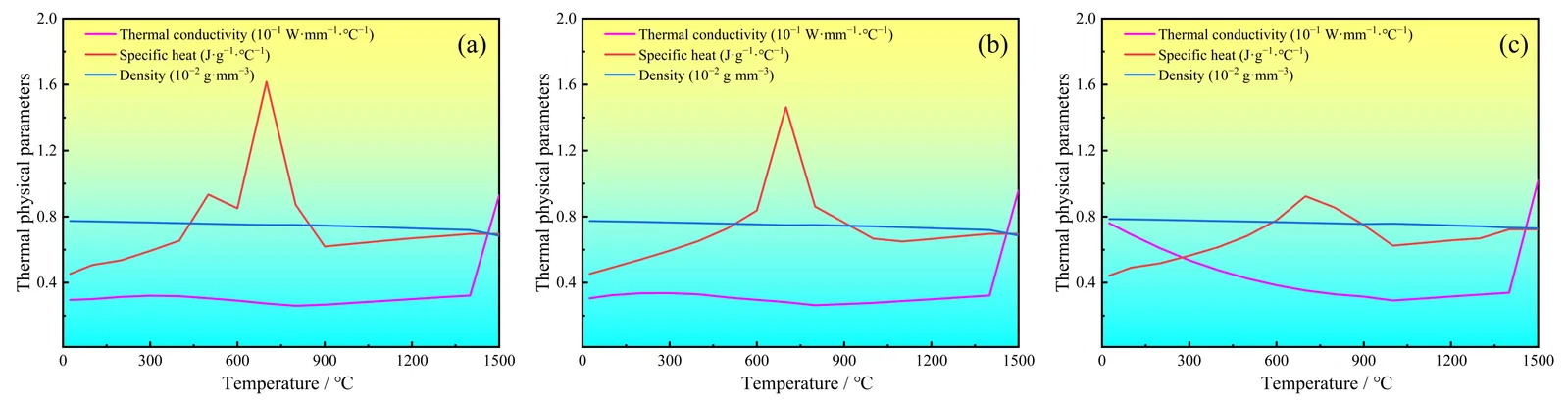
Thermal parameters of the materials: (**a**) QP1180+GA, (**b**) QP980+GI, and (**c**) DC53D+ZAM.

**Figure 3:**
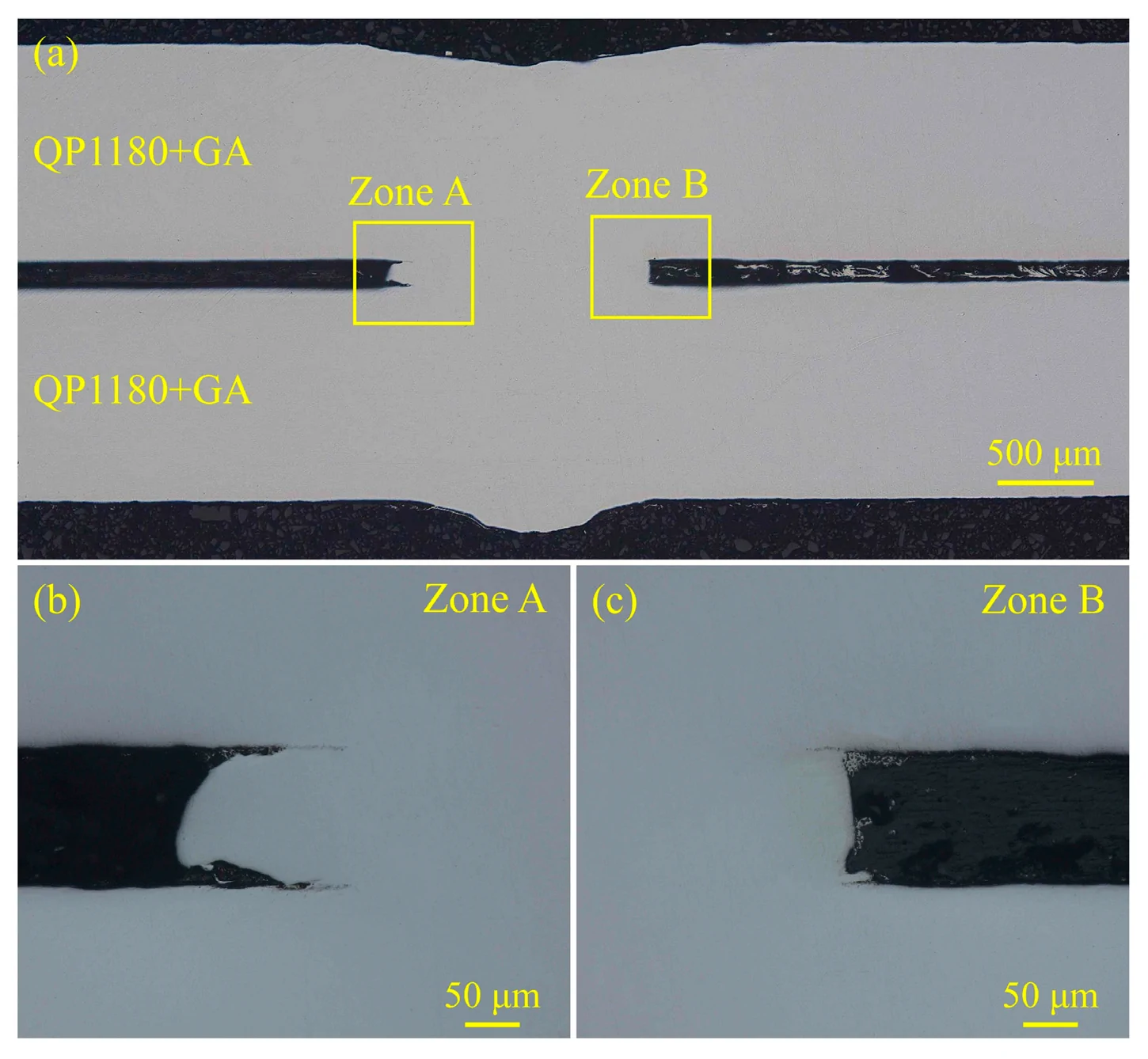
Metallographic images of the unetched Steel A (GA)/Steel A (GA) laser lap-welded joint: (**a**) the overall microstructure of the GA welded joint, (**b**) sheet gap region of Zone A on one side of the joint, and (**c**) sheet gap region of Zone B on the other side of the joint.

**Figure 4:**
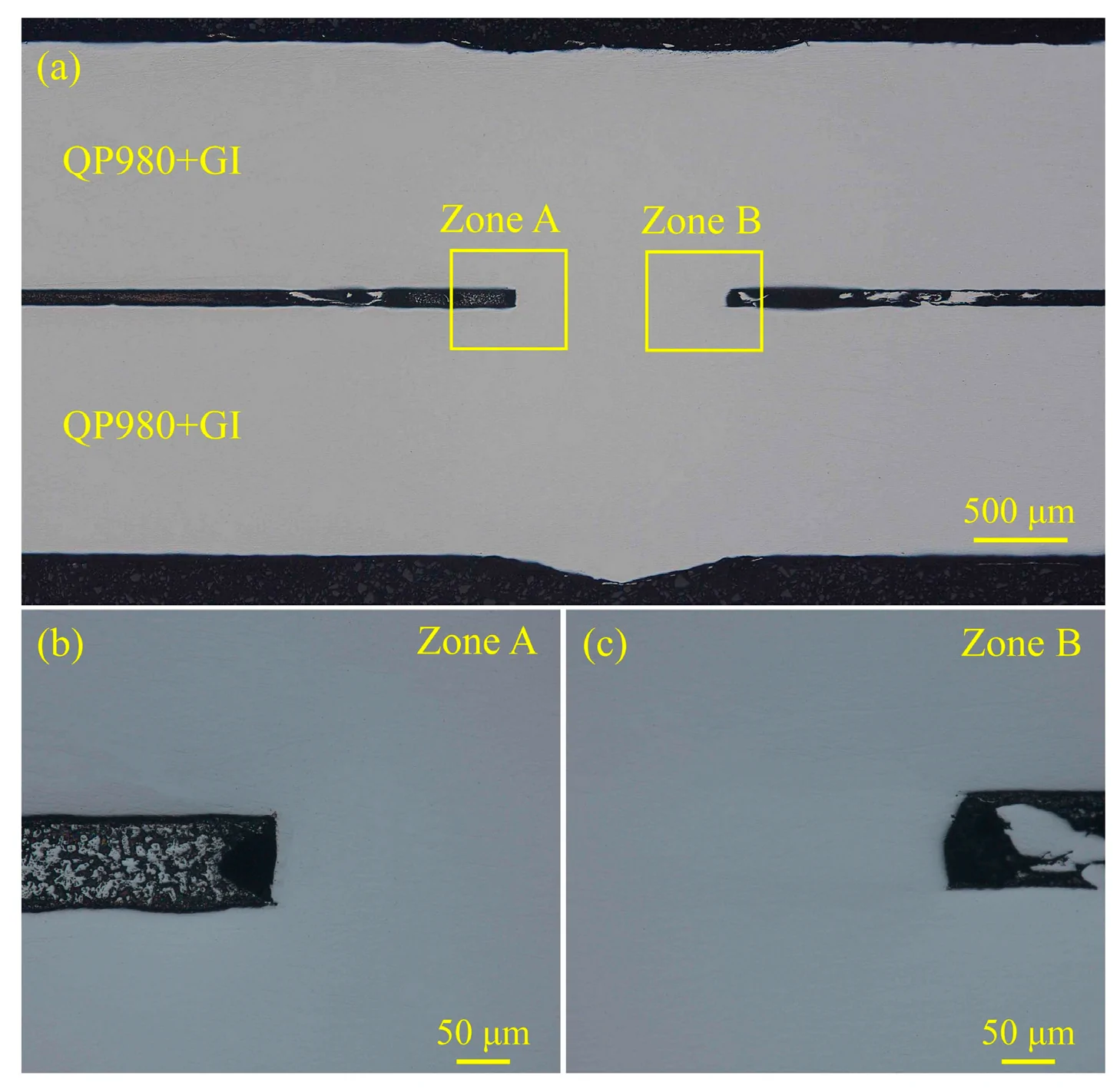
Metallographic images of the unetched Steel B (GI)/Steel B (GI) laser lap-welded joint: (**a**) the overall microstructure of the GI welded joint, (**b**) sheet gap region of Zone A on one side of the joint, and (**c**) sheet gap region of Zone B on the other side of the joint.

**Figure 5:**
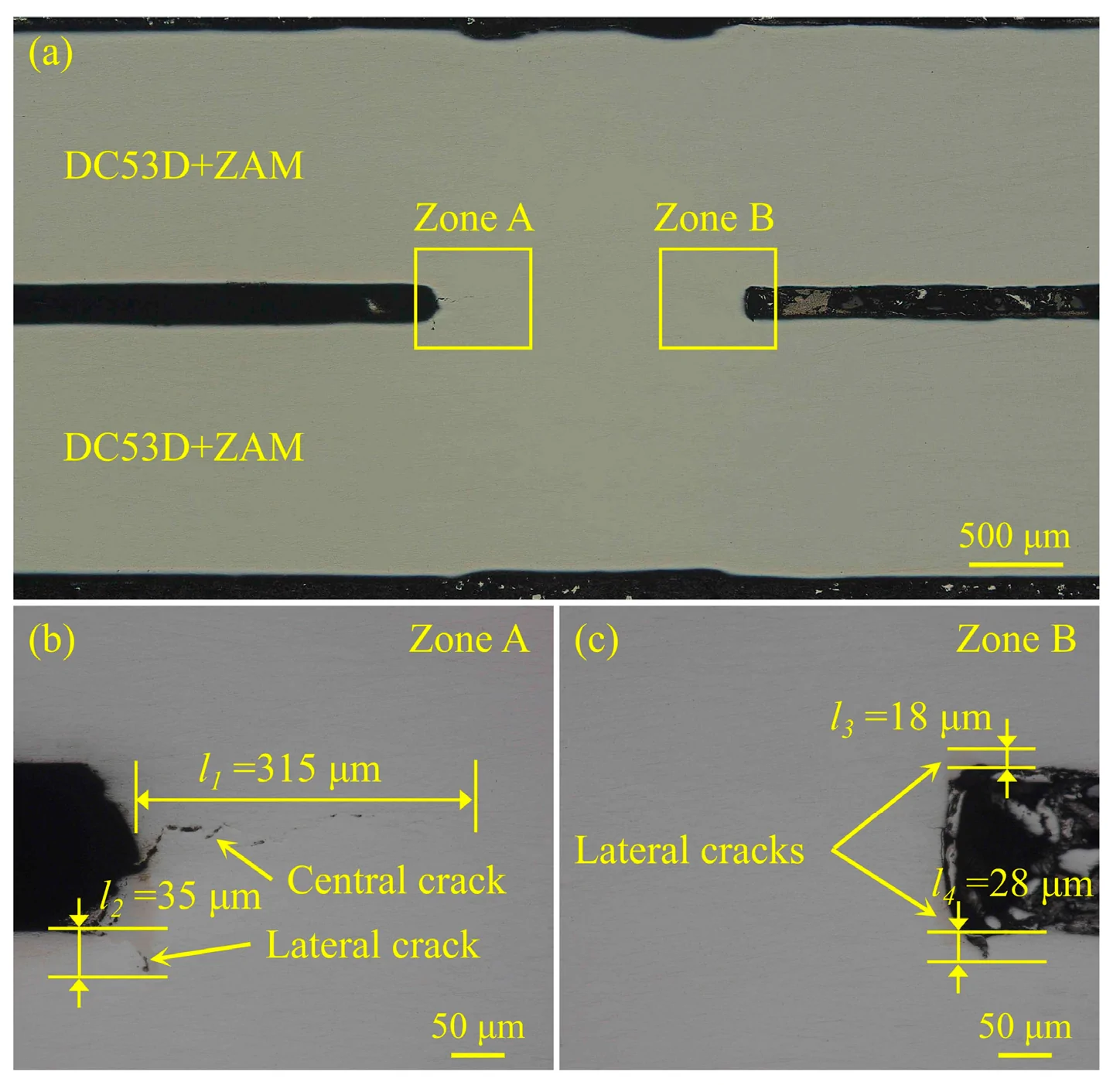
Metallographic images of the unetched Steel C (ZAM)/Steel C (ZAM) laser lap-welded joint: (**a**) the overall microstructure of the ZAM welded joint, (**b**) central crack and lateral crack in the sheet gap of Zone A, and (**c**) lateral crack in the sheet gap of Zone B.

**Figure 6:**
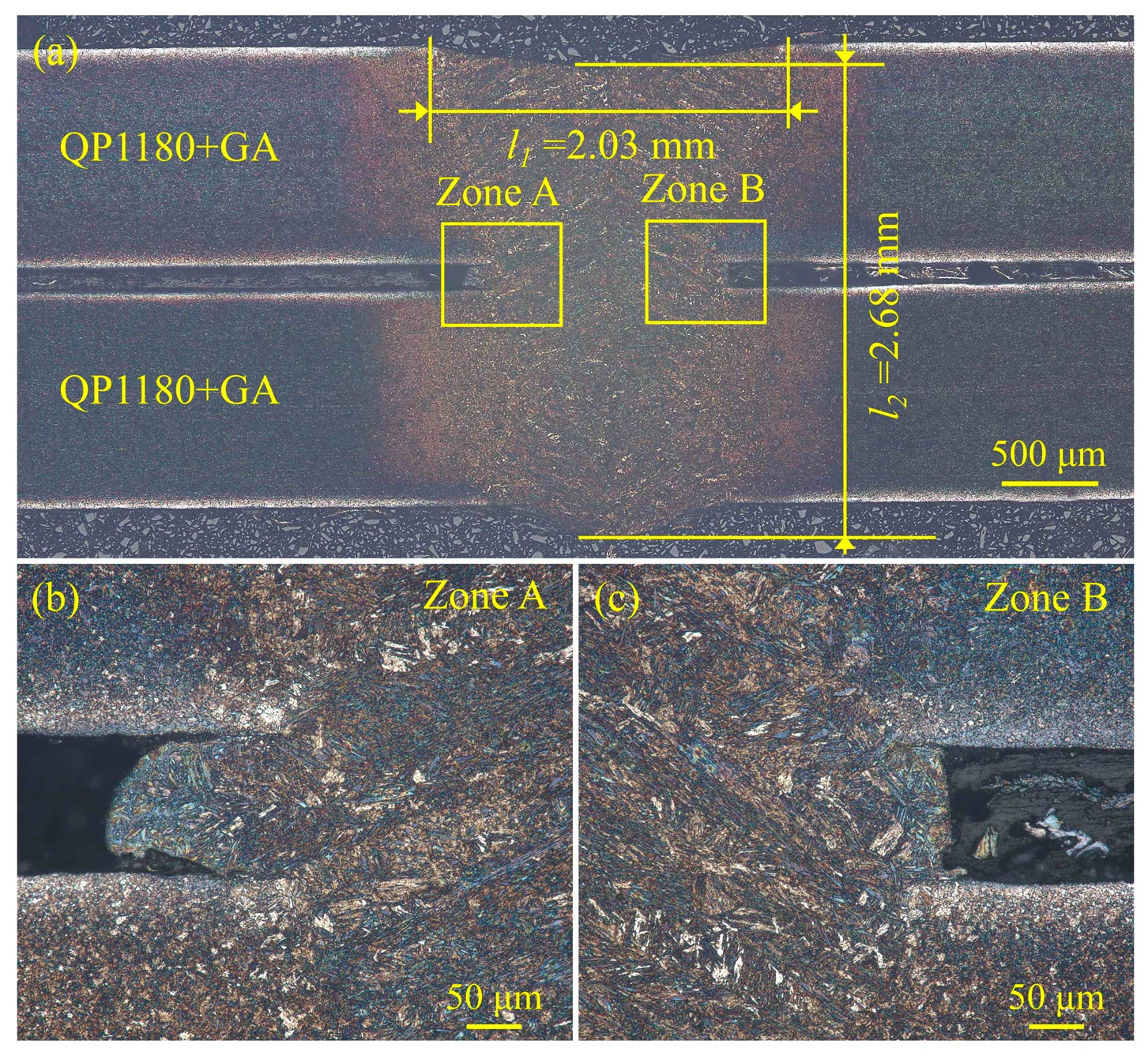
Metallographic images of the etched Steel A (GA)/Steel A (GA) laser lap-welded joint: (**a**) overall microstructure of the GA welded joint, (**b**) sheet gap region of Zone A on one side, and (**c**) sheet gap region of Zone B on the opposite side.

**Figure 7:**
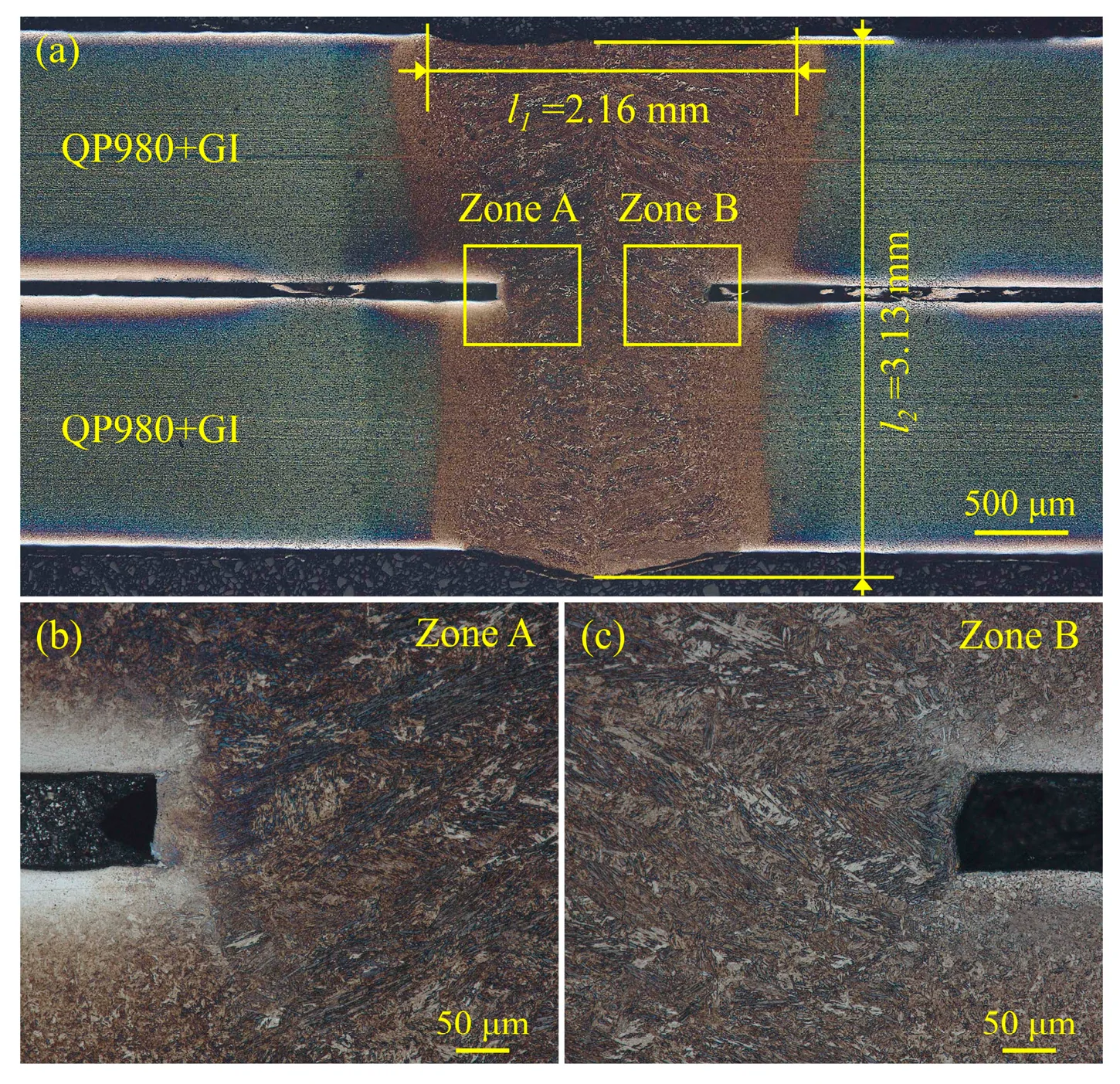
Metallographic images of the etched Steel B (GI)/Steel B (GI) laser lap-welded joint: (**a**) overall microstructure of the GI welded joint, (**b**) sheet gap region of Zone A on one side, and (**c**) sheet gap region of Zone B on the opposite side.

**Figure 8:**
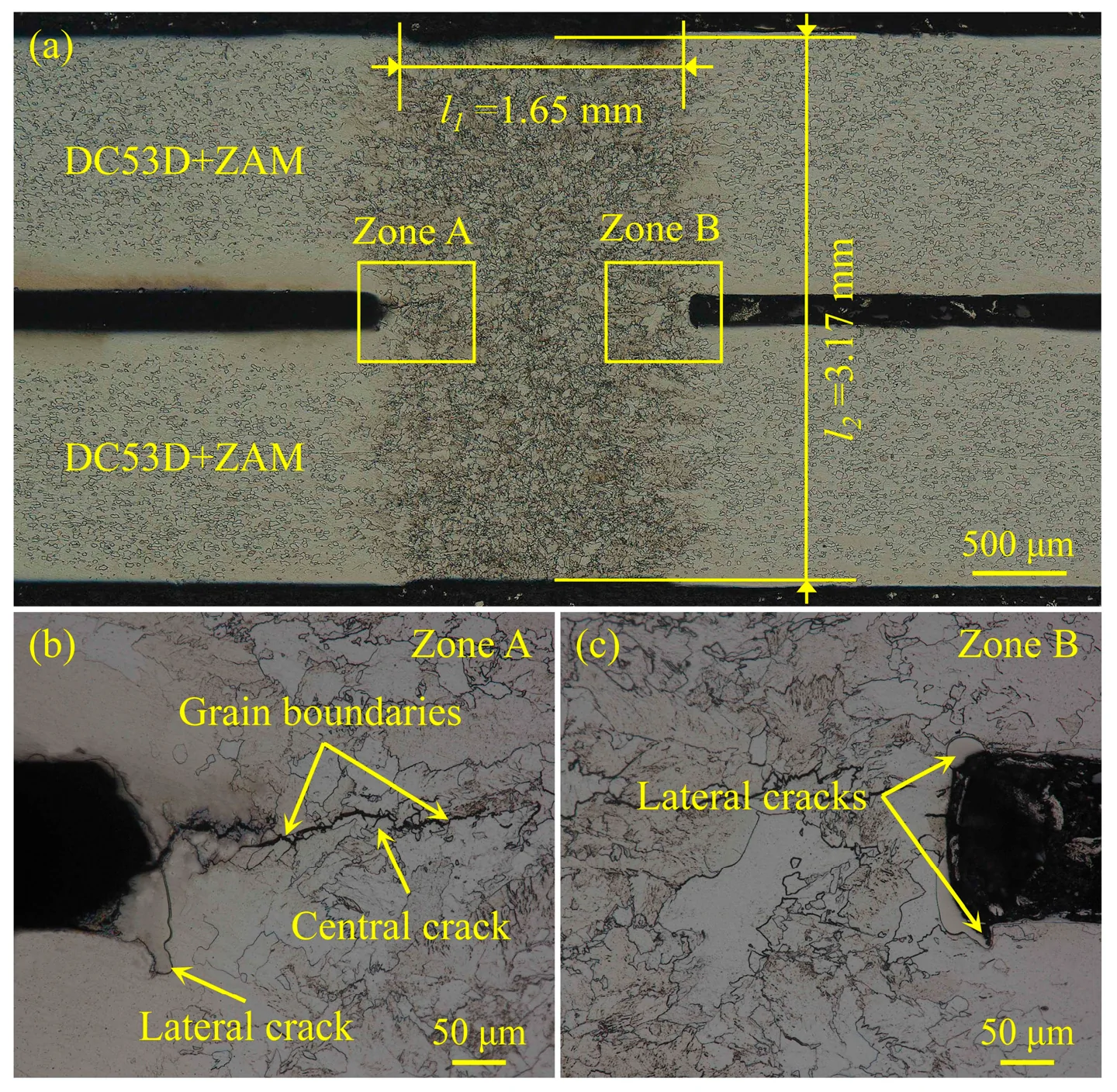
Metallographic images of the etched Steel C (ZAM)/Steel C (ZAM) laser lap-welded joint: (**a**) the overall microstructure of the ZAM welded joint, (**b**) central crack and lateral crack in the sheet gap of Zone A, (**c**) lateral crack in the sheet gap of Zone B.

**Figure 9:**
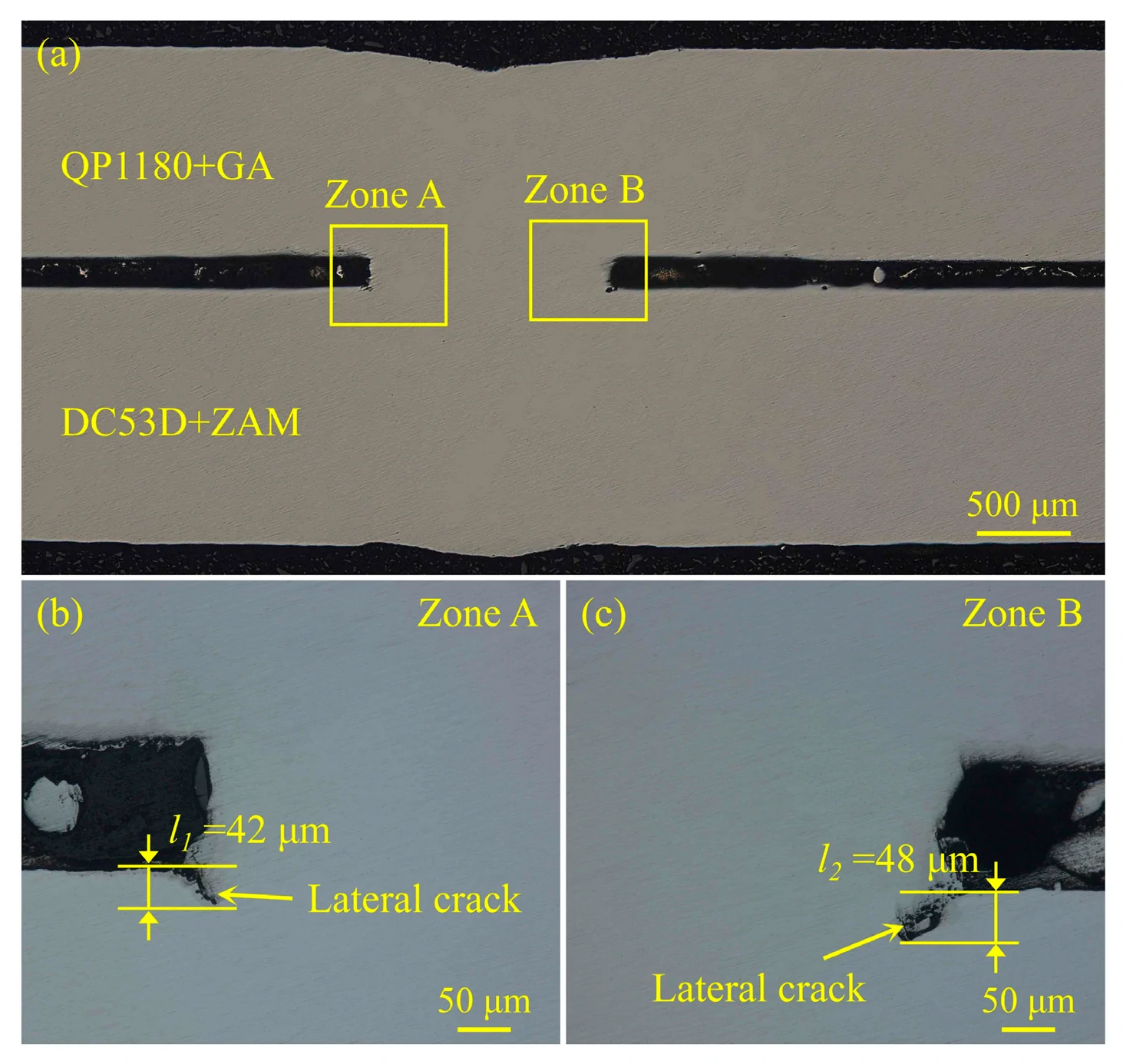
Metallographic images of the unetched Steel A (GA)/Steel C (ZAM) laser lap-welded joint: (**a**) the overall microstructure of the GA+ZAM dissimilar coated steel welded joint, (**b**) lateral crack in the sheet gap of region A, (**c**) lateral crack in the sheet gap of region B.

**Figure 10:**
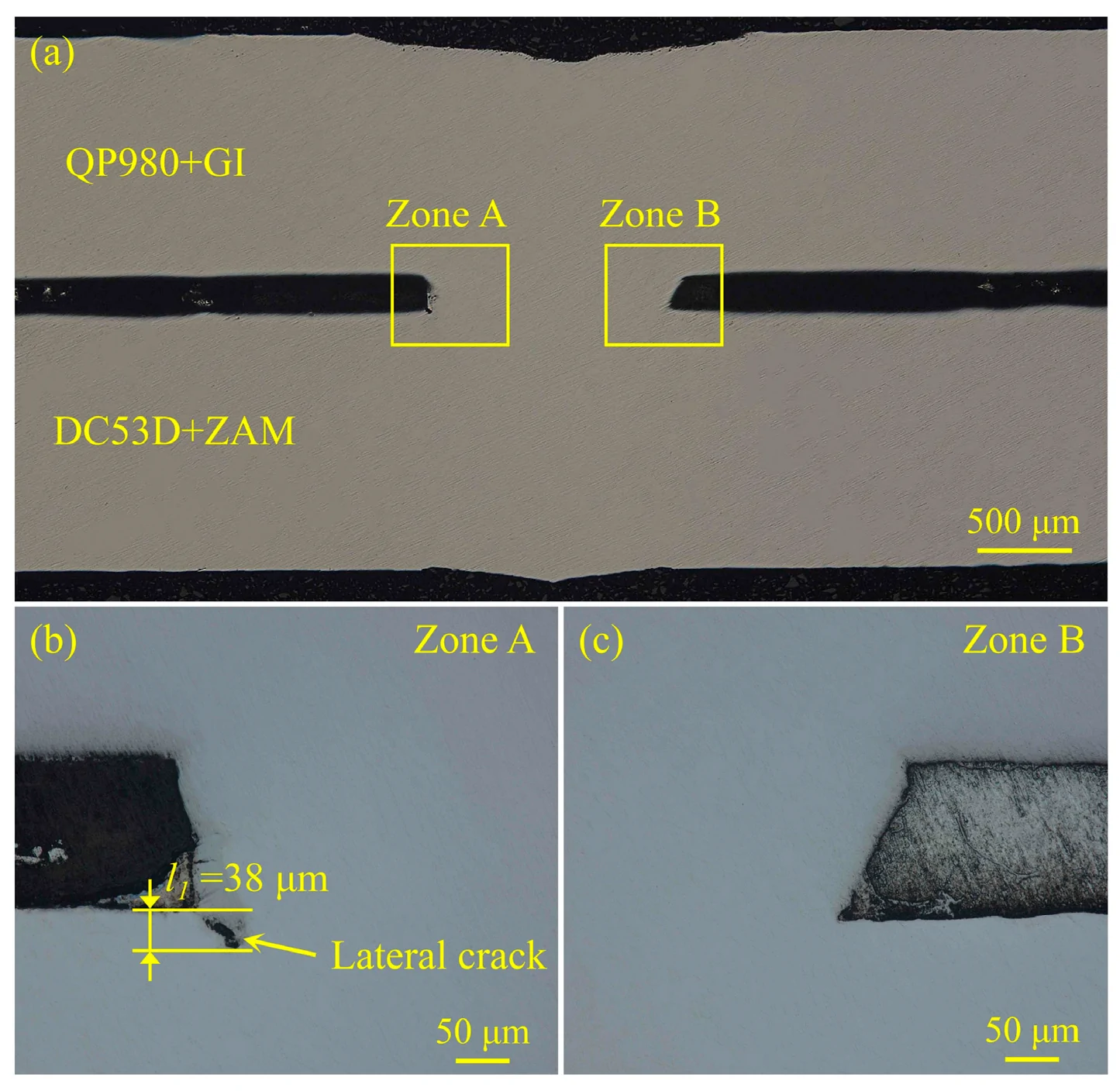
Metallographic images of the unetched Steel B (GI)/Steel C (ZAM) laser lap-welded joint: (**a**) the overall microstructure of the GI+ZAM dissimilar coated steel welded joint, (**b**) lateral crack in the sheet gap of Zone A, and (**c**) sheet gap area on the other side of Zone B.

**Figure 11:**
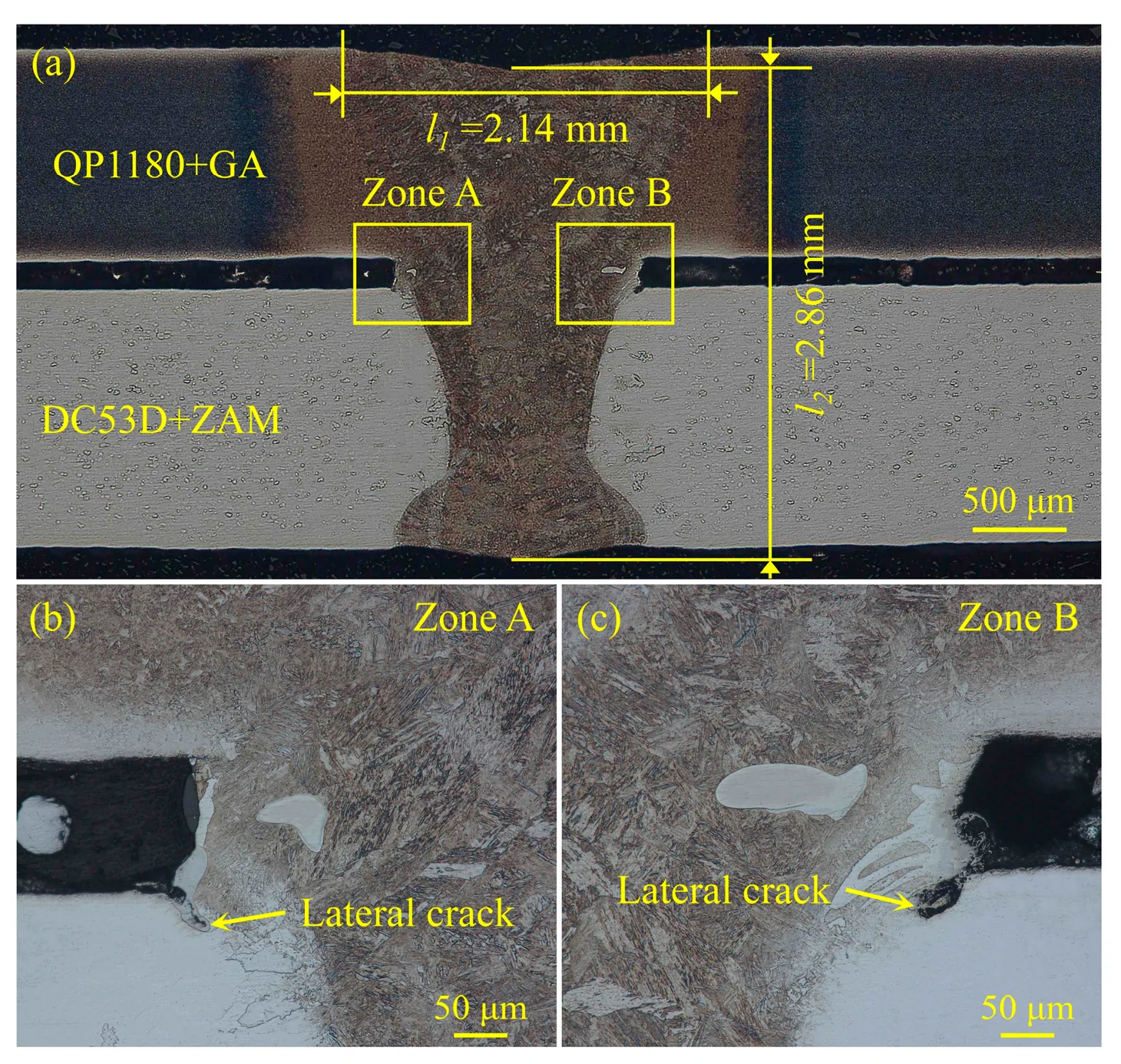
Metallographic images of the etched Steel A (GA)/Steel C (ZAM) laser lap-welded joint: (**a**) the overall microstructure of the GA+ZAM dissimilar coated steel welded joint, (**b**) lateral crack in the sheet gap of Zone A, (**c**) lateral crack in the sheet gap of Zone B.

**Figure 12:**
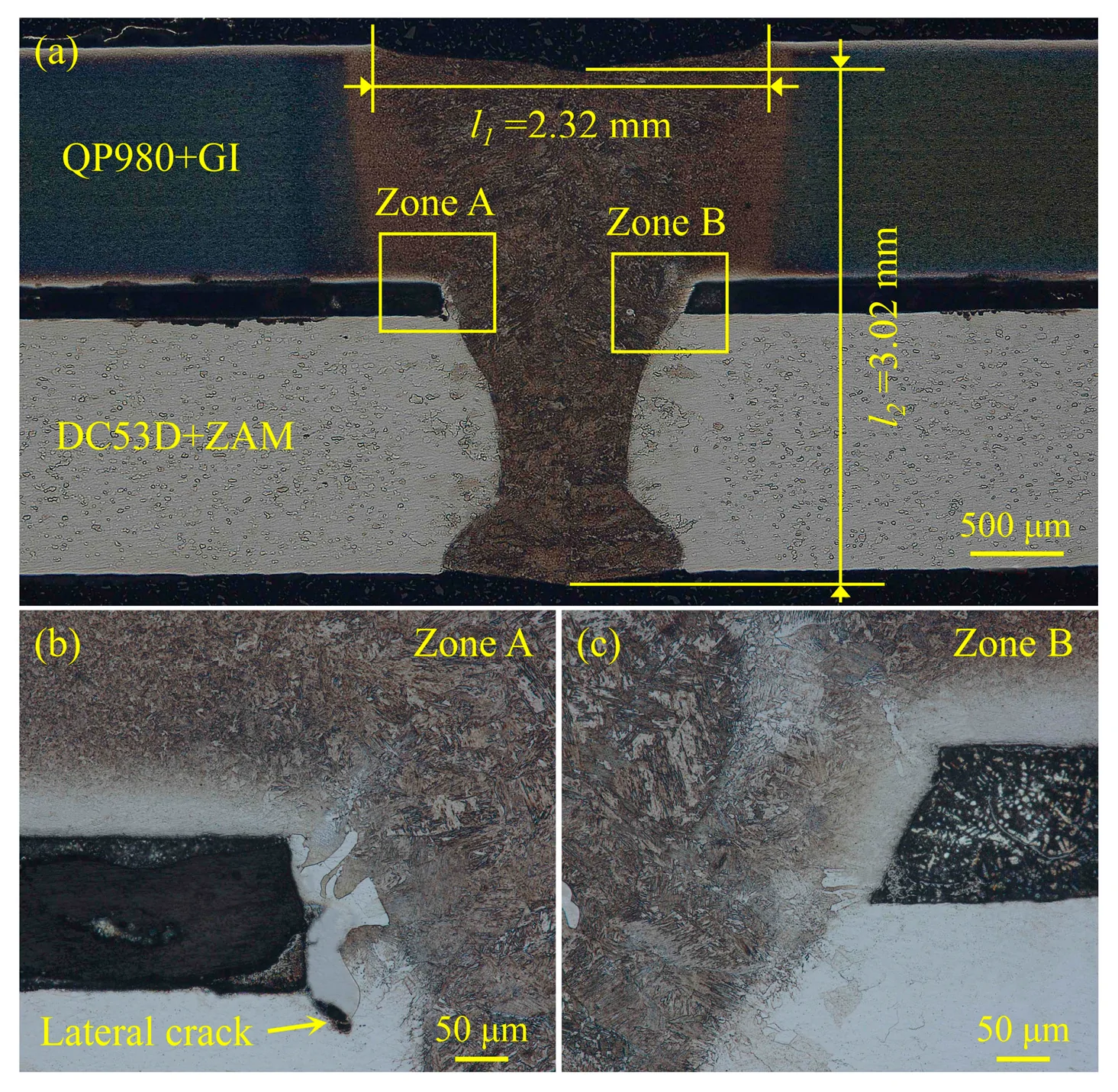
Metallographic images of the etched Steel B (GI)/Steel C (ZAM) laser lap-welded joints: (**a**) the overall microstructure of the GI+ZAM dissimilar coated steel welded joint, (**b**) lateral crack in the sheet gap of Zone A, and (**c**) sheet gap area on the other side of Zone B.

**Figure 13:**
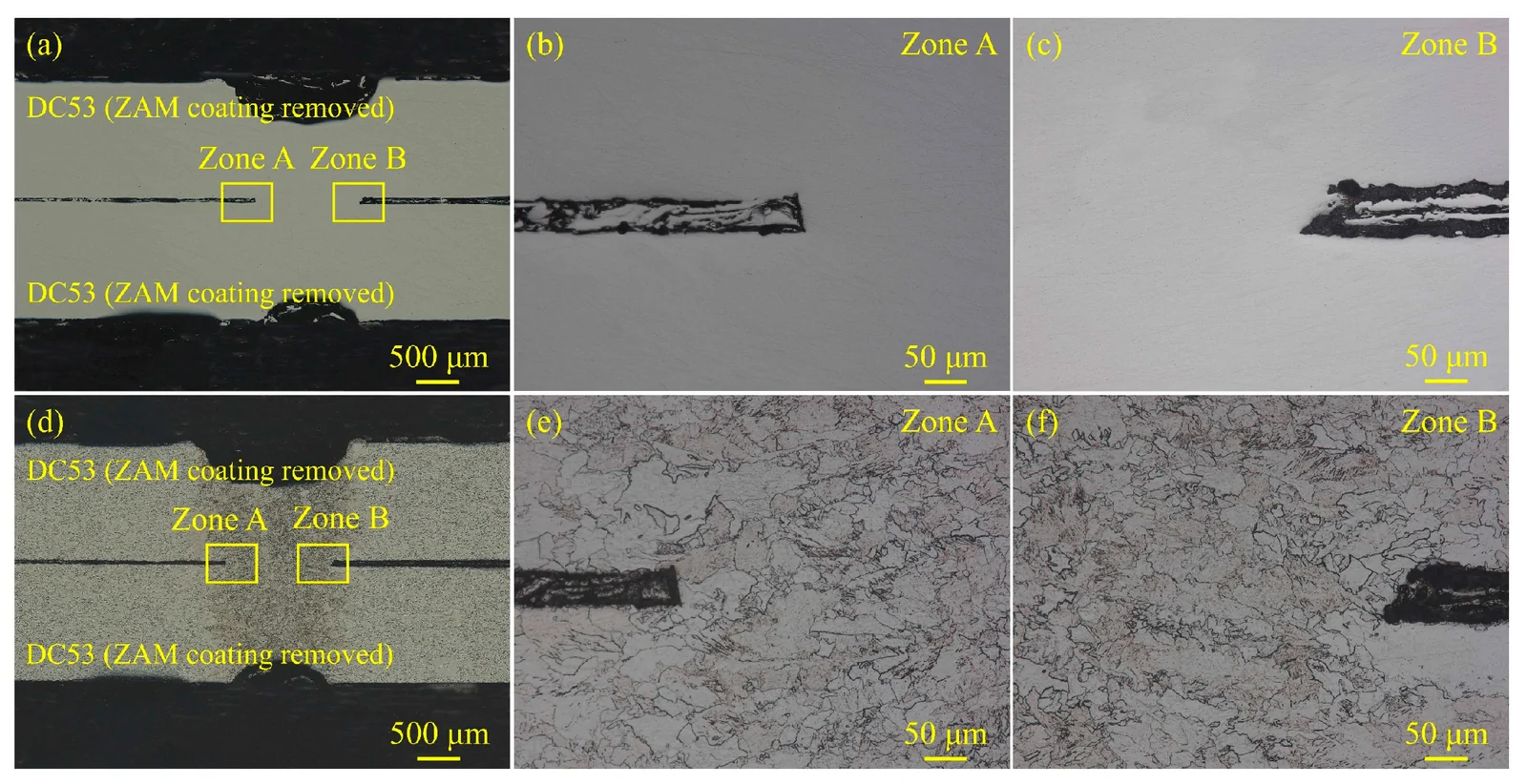
Metallographic images of the unetched (**a**–**c**) and etched (**d**–**f**) DC53 (ZAM coating removed)/DC53 (ZAM coating removed) laser lap-welded joints.

**Figure 14:**
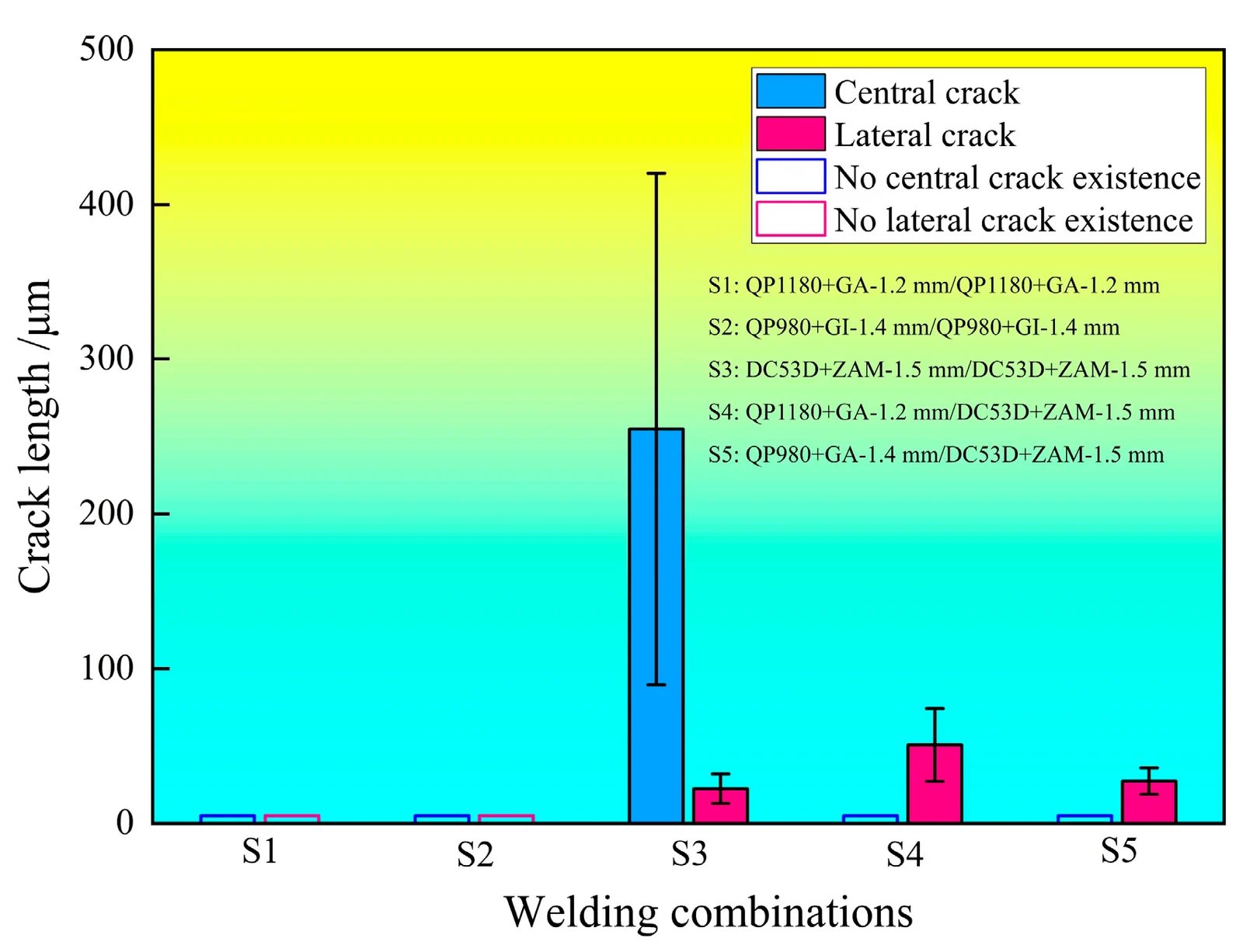
Statistical results of the measured average crack lengths for S1–S5 welding combinations.

**Figure 15:**
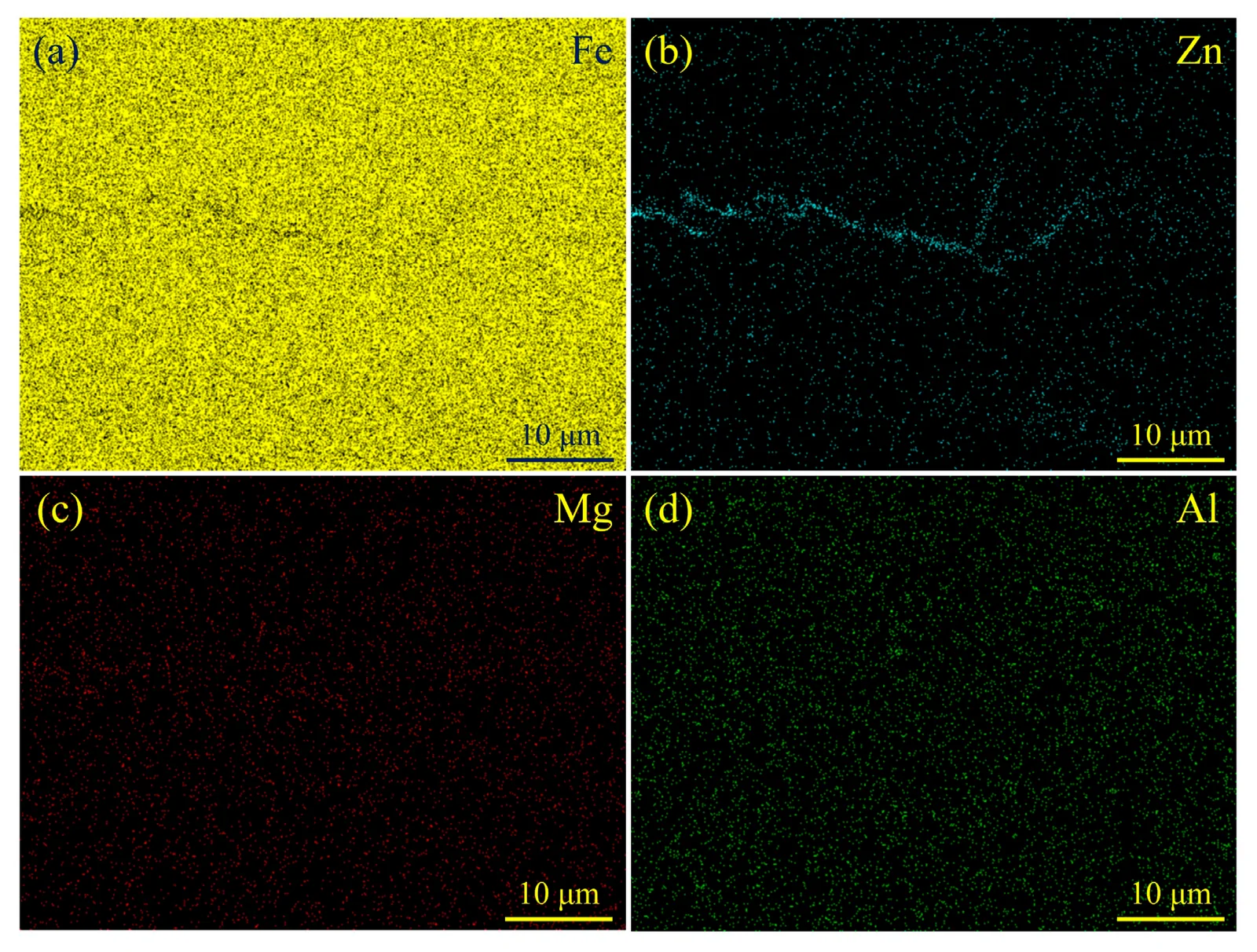
EDS mapping of the elements adjacent to the central crack: (**a**) Fe, (**b**) Zn, (**c**) Mg, (**d**) Al.

**Figure 16:**
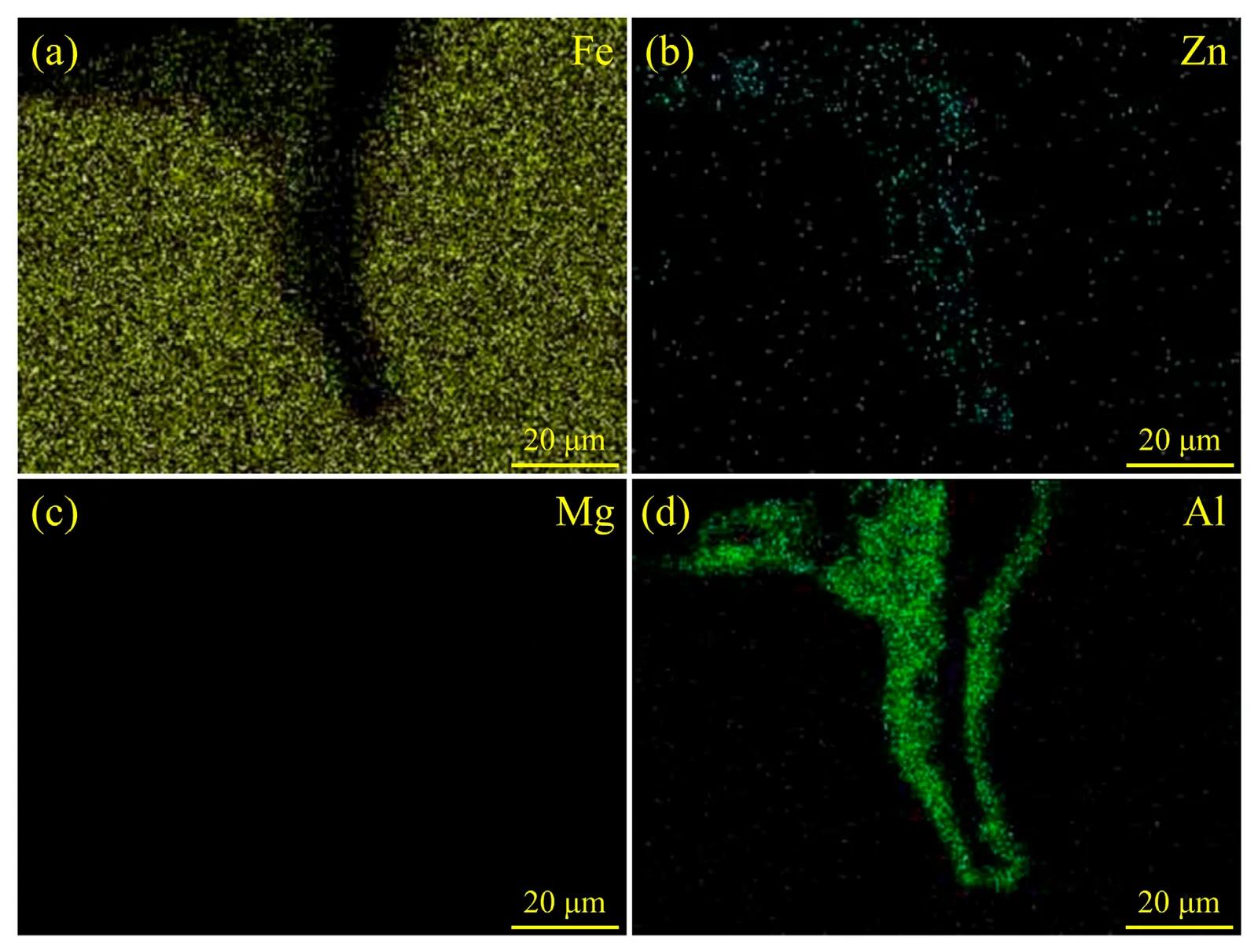
EDS mapping of the elements adjacent to the lateral crack:(**a**) Fe, (**b**) Zn, (**c**) Mg, (**d**) Al.

**Figure 17:**
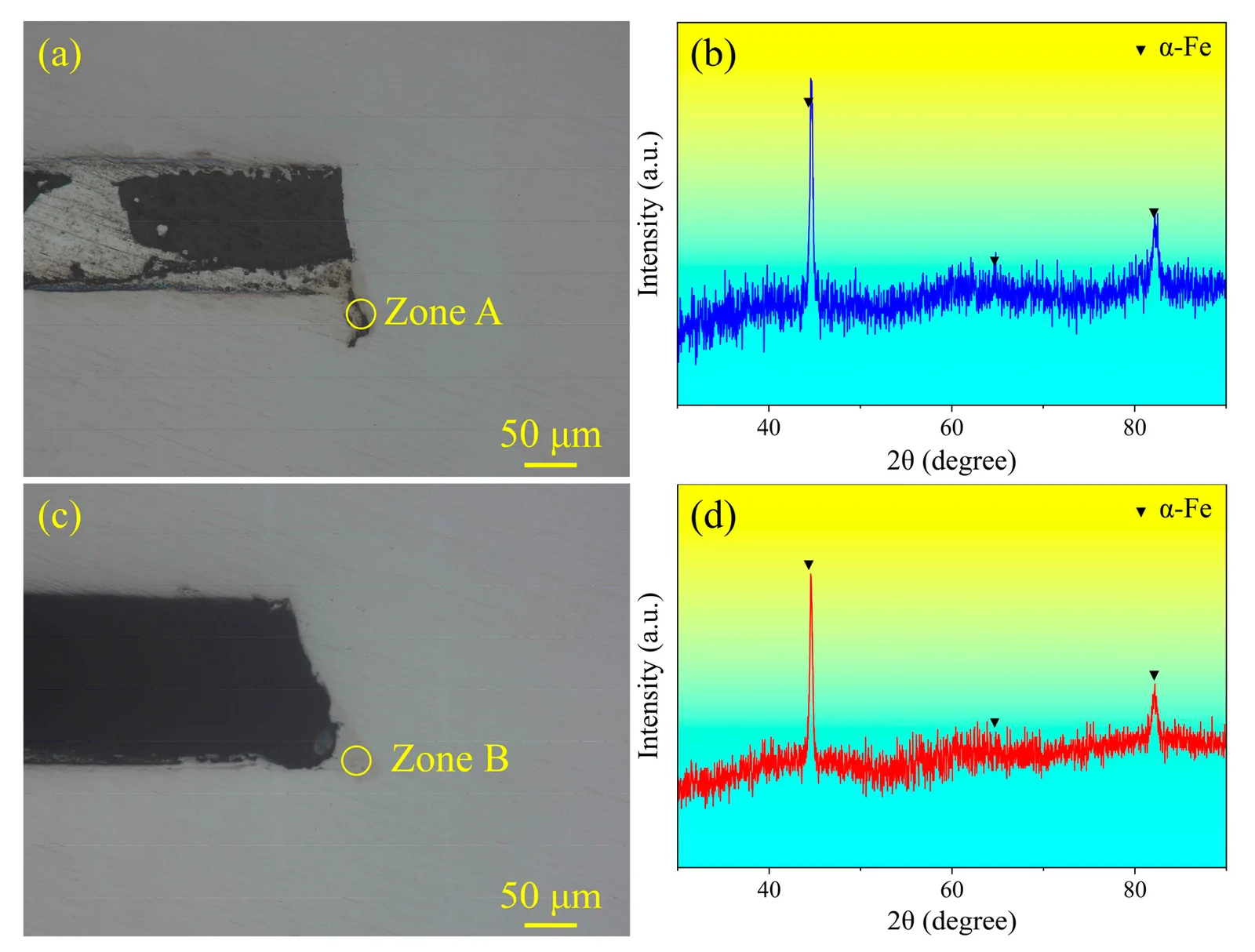
Micro-area XRD test results: (**a**) lateral crack in sample S4, (**b**) micro-area XRD spectrum obtained from Zone A in (**a**), (**c**) lateral crack in sample S5, (**d**) micro-area XRD spectrum obtained from Zone B in (**c**).

**Figure 18:**
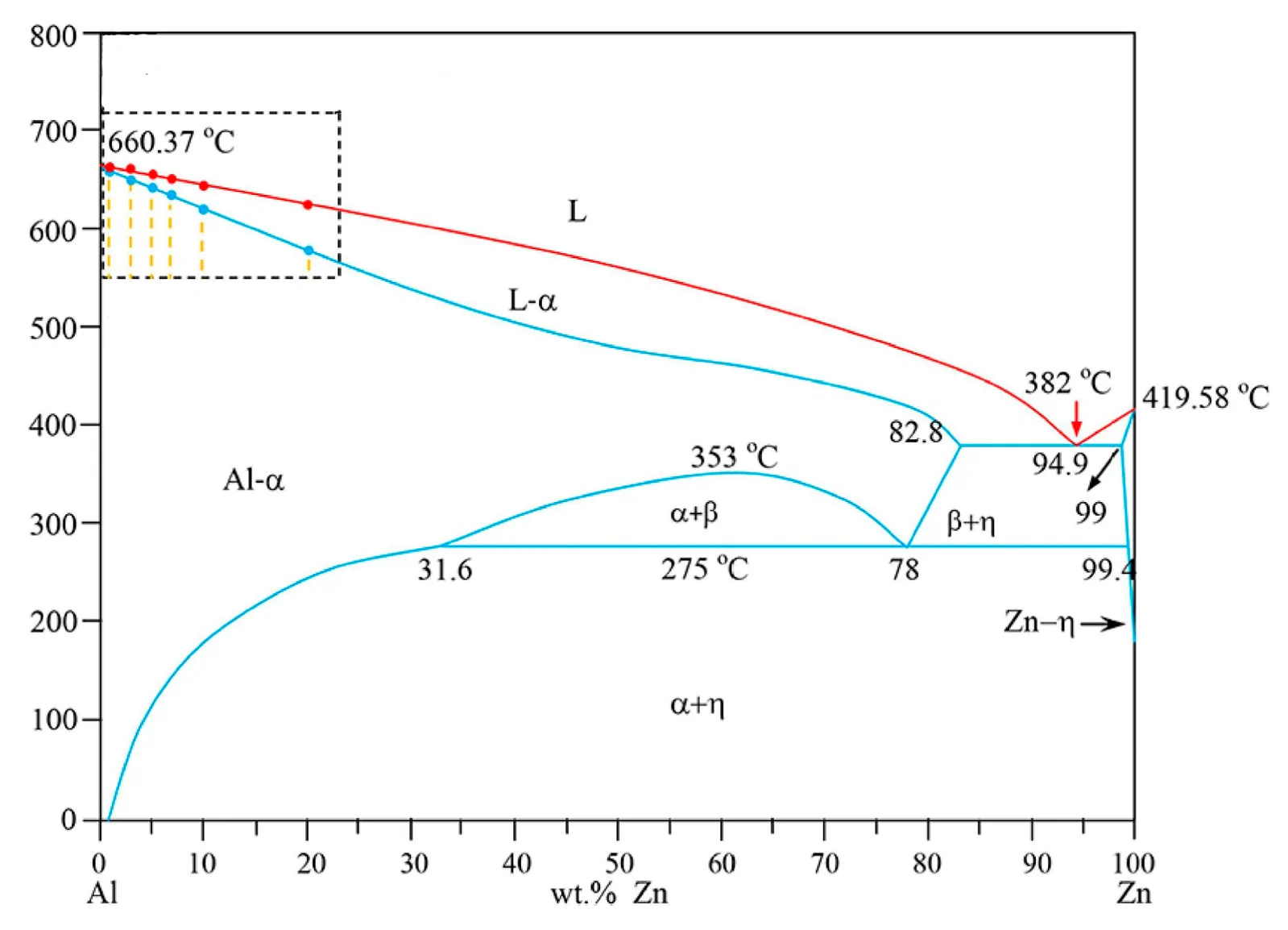
Equilibrium phase diagram of binary Al-Zn system [43].

**Figure 20:**
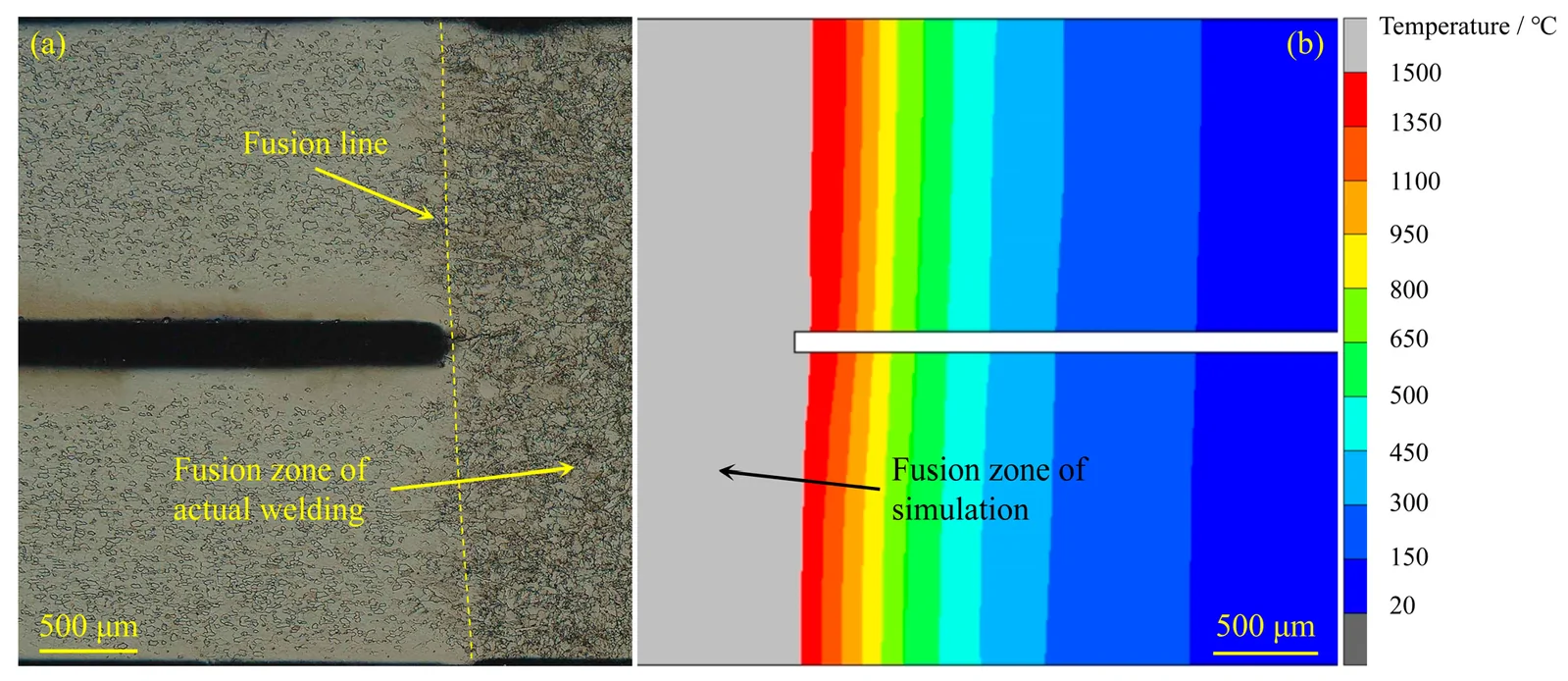
Comparison of the fusion zone between the experimental and numerical simulation results for laser lap-welded joint S3 (DC53D+ZAM/DC53D+ZAM): (**a**) metallographic image of the as-welded half cross-section; (**b**) simulated peak temperature contour plot of the half cross-section.

**Figure 21:**
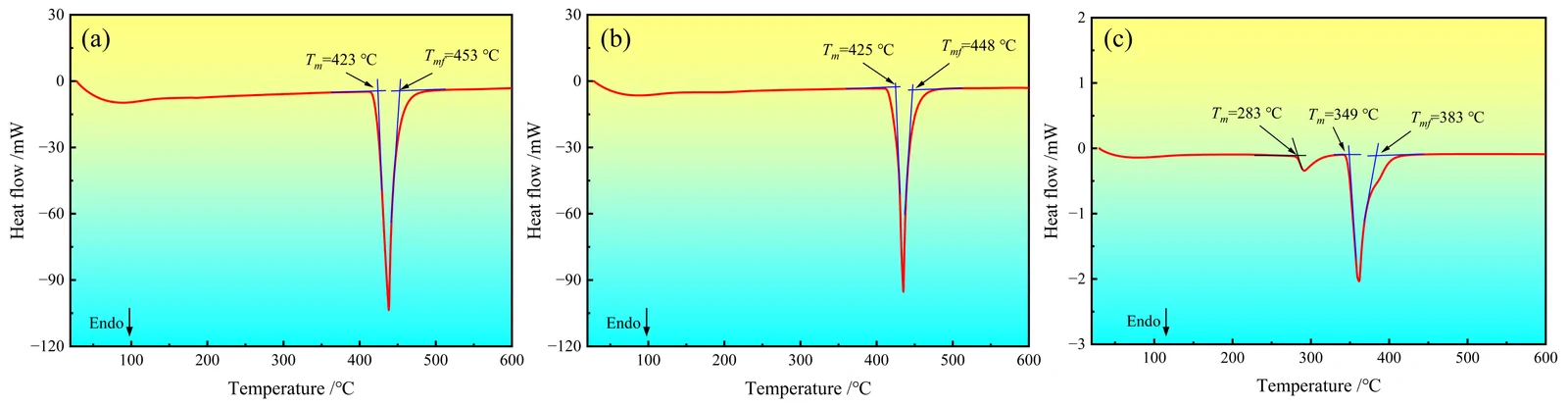
Fast scanning calorimetry (FSC) curves of the coatings: (**a**) GA, (**b**) GI, (**c**) ZAM.

**Figure 22:**
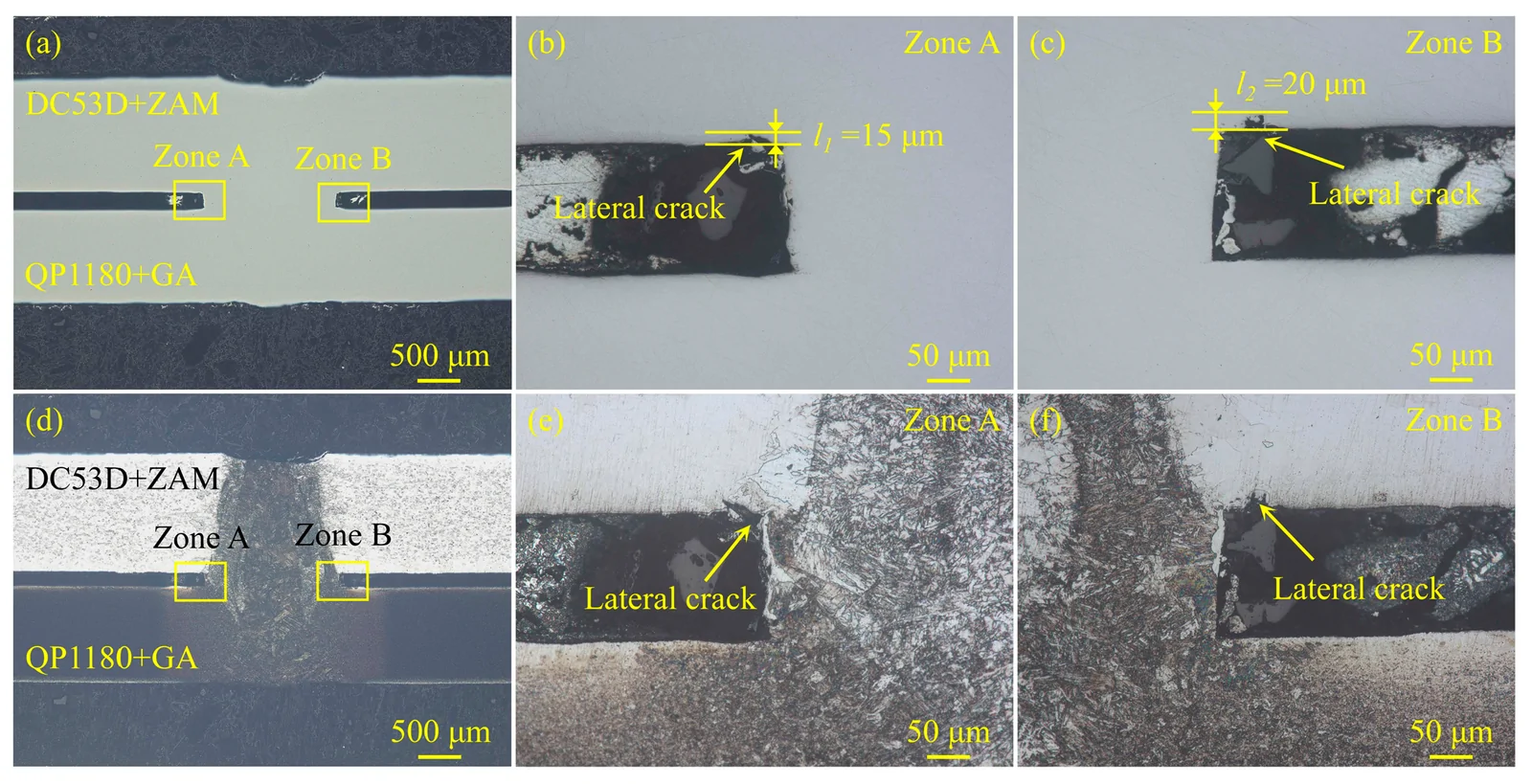
Metallographic images of the unetched (**a**–**c**) and etched (**d**–**f**) DC53D+ZAM/QP1180+GA laser lap-welded joints.

**Figure 23:**
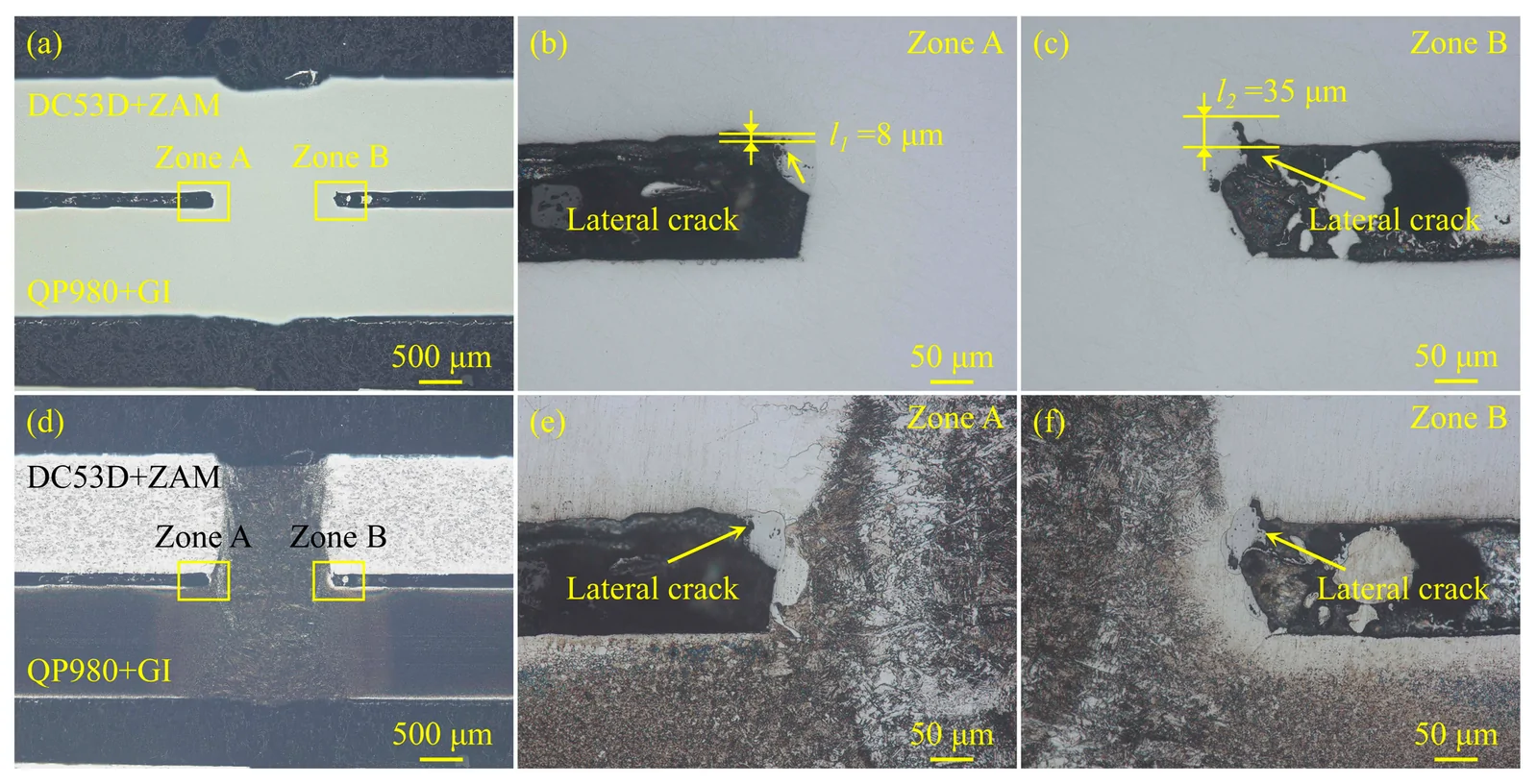
Metallographic images of the unetched (**a**–**c**) and etched (**d**–**f**) DC53D+ZAM/QP980+GI laser lap-welded joints.

**Table 1:** The nominal chemical composition of the different coated steels (wt.%).

Grades	Materials	C	Si	Mn	P	S	Fe
QP1180+GA	Steel A	0.18–0.24	1.6–1.9	2.4–2.8	≤0.015	≤0.02	Bal.
QP980+GI	Steel B	0.16–0.22	1.4–1.8	1.8–2.2	≤0.015	≤0.02	Bal.
DC53D+ZAM	Steel C	0.0005–0.001	0.002–0.01	0.04–0.1	≤0.015	≤0.02	Bal.

**Table 2:** Mechanical properties of the materials used in this study.

Grades	Materials	Yield Strength (MPa)	Tensile Strength (MPa)	Elongation (%)
QP1180+GA	Steel A	975	1222	15.5
QP980+GI	Steel B	705	1017	22.7
DC53D+ZAM	Steel C	162	283	45.7

**Table 3:** Material information of different laser lap welding combinations.

Sample Numbers	Upper Sheet			Lower Sheet		
	Materials	Coatings	Thickness/mm	Materials	Coatings	Thickness/mm
S1	Steel A	GA	1.2	Steel A	GA	1.2
S2	Steel B	GI	1.4	Steel B	GI	1.4
S3	Steel C	ZAM	1.5	Steel C	ZAM	1.5
S4	Steel A	GA	1.2	Steel C	ZAM	1.5
S5	Steel B	GI	1.4	Steel C	ZAM	1.5

## Data Availability

The original contributions presented in this study are included in the article. Further inquiries can be directed to the corresponding authors.

## References

[1] Wang J. (2026). Advances in the understanding of corrosion and performance of hot-dip galvanized rebar in concrete structures. Corros. Rev..

[2] Amar S., Conforto E., Kepa T., Serjaouan M., Creus J., Touzain S., Refait P., Pedraza F. (2026). In-situ marine corrosion experiments during extended immersion: Metallic coatings deposited onto mild steel. Mater. Today Commun..

[3] Prosek T., Nazarov A., Goodwin F., Šerák J., Thierry D. (2016). Improving corrosion stability of ZnAlMg by alloying for protection of car bodies. Surf. Coat. Technol..

[4] Chaouki A., Ali M.B., El Maalam K., Aouadi K., Benabdallah I., El Fatimy A., Naamane S. (2024). Optimizing corrosion protection: Performance comparison of Zn and Zn-Al-Mg alloys hot-dip galvanized coatings. J. Alloys Compd..

[5] Chakraborty A., Ghosh R., Sudan M., Mondal A. (2022). Improvement in hot dip galvanized coating microstructure and properties by pre-metallic deposition on steel surface: A comprehensive review. Surf. Coat. Technol..

[6] Choi J., Jung E.J., Park D.J., Kim H.W., Huh J.Y., Kim T.C., Kim M.S. (2024). Impact of cooling rates on the microstructure and cracking susceptibility of hot-dip galvanized Zn-12wt% Al-5wt% Mg coatings. Surf. Coat. Technol..

[7] Kania H. (2022). Structure and corrosion resistance of coatings obtained by the batch double hot dip method in eutectoid ZnAl bath with the addition of Mg and Si. Coatings.

[8] de Azevedo Alvarenga E., de Freitas Cunha Lins V. (2016). Atmospheric corrosion evaluation of electrogalvanized, hot-dip galvanized and galvannealed interstitial free steels using accelerated field and cyclic tests. Surf. Coat. Technol..

[9] Liu Z.B., Li J.X., Peng H.P., Xie A.J., Li Z.W. (2023). Effect of Mg addition on microstructure and sacrificial anode protection performance of hot dip Al-5Zn-4Si-xMg coating. Coatings.

[10] Kim S.H., Jin S.Y., Yang J.H., Yang J.H., Lee M.H., Yun Y.S. (2024). Self-healing phenomenon at the cut edge of Zn-Al-Mg alloy coated steel in chloride environments. Coatings.

[11] Bakhsheshi-Rad H.R., Hamzah E., Low H.T., Kasiri-Asgarani M., Farahany S., Akbari E., Cho M.H. (2017). Fabrication of biodegradable Zn-Al-Mg alloy: Mechanical properties, corrosion behavior, cytotoxicity and antibacterial activities. Mater. Sci. Eng. C.

[12] So S.M., Grandhi S., Kwon E.P., Oh M.S. (2024). Effects of Si addition on interfacial microstructure and corrosion resistance of hot-dip Zn-Al-Mg-Si alloy-coated steel. Crystals.

[13] Kim S.H., Kang Y.J., Lee K.H., Kang J., Lee M.H., Yun Y.S. (2024). Analysis of blackening reaction of Zn-Mg-Al alloy-coated steel prepared by water vapor treatment. Coatings.

[14] Lu X.Q., Wang S.R., Xiong T.Y., Wen D.S., Wang G.Q., Du H. (2019). Study on corrosion resistance and wear resistance of Zn-Al-Mg/ZnO composite coating prepared by cold spraying. Coatings.

[15] He C., Wang Z.J., You Y., Wang X.X., Zhao P.Z., Yang Z.S., Zhou L.F. (2025). Influence of soil variability on the corrosion of buried hot-dip galvanized steel. Int. J. Electrochem. Sci..

[16] Liu Y.S., Gao H.Y., Wang H., Tao X., Zhou W.Z. (2024). Study on the corrosion behavior of hot-dip galvanized steel in simulated industrial atmospheric environments. Int. J. Electrochem. Sci..

[17] Okamoto N.L., Inomoto M., Takebayashi H., Inui H. (2018). Crystal structure refinement of the Γ- and Γ1-phase compounds in the Fe-Zn system and orientation relationships among α-Fe, Γ and Γ1 phases in the coating layer of galvannealed steel. J. Alloys Compd..

[18] Shibli S.M.A., Meena B.N., Remya R. (2015). A review on recent approaches in the field of hot dip zinc galvanizing process. Surf. Coat. Technol..

[19] Yang D.H., Wang K.P., Zhou H.W., Zhang X., Zhang B., Lu B.Z., Wang F. (2023). Microstructure and properties of galvannealed coatings at different galvannealed time. Mater. Lett..

[20] Li Z., Li Y.P., Jiang S.M., Zhang J., Liu X., Zhang Q.F., Liu Q.Y. (2024). Calculation and experimental verification of Zn-Al-Mg phase diagram. Coatings.

[21] Xu L., Cui C.Y., Lu Q., Yang H., Zhang W.Y. (2023). Characterization of microstructural and corrosion behavior of cold sprayed Zn_11_Al_3_Mg alloy coating. Surf. Coat. Technol..

[22] Said V.H., Haakmann F., Brinkbäumer J., Ulbricht M. (2022). Comparison of the nucleation and growth of a phosphate conversion coating on Zn-Al and Zn-Al-Mg coatings under the influence of a corrosion inhibitor film. Surf. Coat. Technol..

[23] Yao C.Z., Lv H.B., Zhu T.P., Zheng W.G., Yuan X.D., Gao W. (2016). Effect of Mg content on microstructure and corrosion behavior of hot dipped Zn-Al-Mg coatings. J. Alloys Compd..

[24] Prosek T., Hagström J., Persson D., Fuertes N., Lindberg F., Chocholatý O., Taxén C., Šerák J., Thierry D. (2016). Effect of the microstructure of Zn-Al and Zn-Al-Mg model alloys on corrosion stability. Corros. Sci..

[25] Yasakau K.A., Kallip S., Lisenkov A., Ferreira M.G.S., Zheludkevich M.L. (2016). Initial stages of localized corrosion at cut-edges of adhesively bonded Zn and Zn-Al-Mg galvanized steel. Electrochim. Acta.

[26] Zhang Z., Yue Z., Li X., Jiang B., Wang F., Fan Z., Yan F., Li W., Wu Y., Mi C. (2020). Microstructure and corrosion resistance of Zn-Al-Mg alloy diffusion coating developed on carbon steel by hot dipping. Int. J. Electrochem. Sci..

[27] Kouadri-Henni A. (2017). Effect of welding laser process on macrostructures and the mechanical properties of coating steel DP600: Influence of vaporization zinc. J. Manuf. Process..

[28] Qi Y., Chen G., Liu D. (2022). Droplet spatter suppression in laser lap welding of galvanized sheets using additional coaxial annular laser source. Opt. Laser Technol..

[29] Bautista-Rodriguez E., García-García V., Reyes-Calderón F., Hernández-Cristóbal O., Baltazar-Hernández V.H. (2025). Mitigation of liquid embrittlement cracks in similar/dissimilar RSW joints between Zn-coated steels DP590 and mild270 through hardening increment: Numerical and experimental analyses. Mater. Today Commun..

[30] Yu Y., Zhang Y., Cai N., Chen W., Wang P., Fu C. (2023). Comparative investigation into the liquid metal embrittlement susceptibility during resistance spot welding of Zn-Al-Mg and GI coated advanced high strength steels. ISIJ Int..

[31] Hu R., Zhou J., Sun Y., Lei M., Gao Y. (2024). Liquid metal embrittlement susceptibility and crack formation of the Zn-coated complex phase steel. Materials.

[32] Zhu Y.J., Wang H.P., Wang Y.Q., Hao Y., Carlson B.E., Lu F.G. (2021). Formation mechanism of liquid metal embrittlement in laser lap welding of zinc-coated GEN3 steels. Mater. Sci. Eng. A.

[33] Dong W.F., Pan H., Lei M., Wang S.J., Ding K., Gao Y.L. (2023). Three-dimension characterization of the liquid metal embrittlement crack in the resistance spot welded joint of the advanced high strength steel. Mater. Today Commun..

[34] Beal C., Kleber X., Fabregue D., Bouzekri M. (2012). Embrittlement of a zinc coated high manganese TWIP steel. Mater. Sci. Eng. A.

[35] Ripperger L., Burlet T., Molodov K.D., Sandlöbes-Haut S. (2025). High temperature compression tests for liquid metal embrittlement characterisation in steels. Mater. Today Commun..

[36] Razmpoosh M.H., Macwan A., Biro E., Chen D.L., Peng Y., Goodwin F., Zhou Y. (2018). Liquid metal embrittlement in laser beam welding of Zn-coated 22MnB5 steel. Mater. Des..

[37] Zhou J.Y., Sun Y., Hu T.H., Pan H., Lei M., Ding K., Gao Y.L. (2024). Uncovering the mechanism of the LME susceptibility for the galvanized and electrogalvanized DP1180 steels. Mater. Today Commun..

[38] Liu C., Su S., Zhang J., Pan H., Gao Y. (2026). Uncover the formation mechanism of the cracks in the laser lap welded steel joints with Zn-Al-Mg coating. Mater. Lett..

[39] Uozumi M., Morisada Y., Ameyama K., Fujii H. (2023). Pressure-controlled linear friction welding of galvanized steel sheets. Q. J. Jpn. Weld. Soc..

[40] Sejč P., Gábrišová Z., Vanko B., Belanová J. (2025). The Influence of different hot-dip zinc coating types on the gas metal arc welding of galvanized steel sheets. J. Mater. Eng. Perform..

[41] Yan X., Yang X., Hu W., Lei Y. (2015). Microstructure, thermal behavior, and wettability of Zn4Al3Mg-xIn solders. J. Mater. Sci. Mater. Electron..

[42] Kageyama Y., Morita K. (2023). Compositional and thermophysical study of Al-Si and Zn-Al-Mg based eutectic alloys for latent heat storage. High Temp. Mater. Process..

[43] Acer E., Cadirli E., Erol H., Kaya H., Sahin M., Gündüz M. (2018). Effect of growth velocity and Zn content on microhardness in directionally solidified Al-Zn alloys. Mater. Res..

[44] Gogola P., Gabalcova Z., Kusy M., Suchanek H. (2021). The effect of Sn addition on Zn-Al-Mg alloy; Part I: Microstructure and phase composition. Materials.

